# Bayesian Analysis of Aberrant Response and Response Time Data

**DOI:** 10.3389/fpsyg.2022.841372

**Published:** 2022-04-25

**Authors:** Zhaoyuan Zhang, Jiwei Zhang, Jing Lu

**Affiliations:** ^1^School of Mathematics and Statistics, Yili Normal University, Yining, China; ^2^Institute of Applied Mathematics, Yili Normal University, Yining, China; ^3^School of Mathematics and Statistics, Yunnan University, Kunming, China; ^4^Key Laboratory of Applied Statistics of MOE, School of Mathematics and Statistics, Northeast Normal University, Changchun, China

**Keywords:** aberrant responses, Bayesian inference, mixture hierarchical model, Pólya-gamma distribution, rapid guessing behavior, Gibbs sampling algorithm

## Abstract

In this article, a highly effective Bayesian sampling algorithm based on auxiliary variables is proposed to analyze aberrant response and response time data. The new algorithm not only avoids the calculation of multidimensional integrals by the marginal maximum likelihood method but also overcomes the dependence of the traditional Metropolis–Hastings algorithm on the tuning parameter in terms of acceptance probability. A simulation study shows that the new algorithm is accurate for parameter estimation under simulation conditions with different numbers of examinees, items, and speededness levels. Based on the sampling results, the powers of the two proposed Bayesian assessment criteria are tested in the simulation study. Finally, a detailed analysis of a high-state and large-scale computerized adaptive test dataset is carried out to illustrate the proposed methodology.

## 1. Introduction

In educational psychological assessments, examinees often perform different types of test-taking behaviors (Bolt et al., 2002; Boughton and Yamamoto, 2007; Goegebeur et al., 2008; Chang et al., 2014; Wang and Xu, 2015; Wang et al., 2018; Man et al., 2018; Man and Harring, 2021). One is the solution behavior, in which the examinee gives a response after careful consideration to each part of an item (Schnipke and Scrams, 1997; Bolt et al., 2002; Wise and Kong, 2005; Wang and Xu, 2015). An alternative is the rapid guessing behavior, in which the examinee simply seeks to obtain an answer quickly without a deep thought process; this behavior often occurs in high-stakes tests owing to insufficient time and in low-stakes tests owing to lack of motivation. In fact, the traditional item response theory (IRT) model is based on the assumption that the correct response probability increases with the ability of the test taker under the solution behavior. The correct response probability under the rapid guessing behavior is actually rarely dependent on the measure constructed by the test (Lord and Novick, 1968; Wise and DeMars, 2006; Boughton and Yamamoto, 2007; Goegebeur et al., 2008). Numerous studies have shown that the presence of rapid guessing behavior inevitably leads to biased inferences of the item and person parameters (Bolt et al., 2002; Wise and DeMars, 2006; Boughton and Yamamoto, 2007; Goegebeur et al., 2008; Chang et al., 2014; Wang and Xu, 2015; Wang et al., 2018). Therefore, appropriate models need to be constructed to capture both solution behavior and rapid guessing behavior to reduce these biased parameter estimates. Before we analyze aberrant response behavior, we provide an explanation of the change point, which is the cut-off point at which an examinee adopts different response strategies. By considering a change point, Bolt et al. (2002) classified examinees in the speeded group before the change point and found that they were more likely to adopt the solution behavior, whereas examinees who transferred from the speeded group to the non-speeded group after the change point were more likely to choose the rapid guessing behavior. In contrast to models using fixed change point locations, Boughton and Yamamoto (2007) proposed the more flexible HYBRID model, which allowed different examinees to have change points at different locations. The model assumes that examinees' responses follow a Rasch model until a particular point in a given examinee's test, after which the responses to all items are randomly guessed. Goegebeur et al. (2008) proposed a speeded model with one change point to characterize the gradual switch between response strategies by introducing an additional examinee-specific change-rate parameter. In addition, Wise and DeMars (2006) proposed an effort-moderated IRT model to decompose the correct response probability into a mixture of two sub-models. The two sub-models were used to characterize the solution behavior and rapid guessing behavior, respectively.

In parallel with the abovementioned item response data, response time, which is an important type of important auxiliary information, has been widely used to distinguish between two different behaviors (Schnipke and Scrams, 1997; Wise and DeMars, 2006; van der Linden and Guo, 2008; Wang and Xu, 2015). van der Linden and Guo (2008) found that examinees' response times in a high-stakes achievement test showed a mixture of two different distributions. Similarly, Schnipke and Scrams (1997) verified that the distribution of response times for end-of-test items showed a bimodal pattern in a high-stakes exam. In the study of (Schnipke and Scrams, 1997), a two-state mixture model was proposed to decompose the distribution of response times for each item into two parts. The two parts of the response times quantified the solution behavior and the rapid guessing behavior, respectively. Wang and Xu (2015) proposed a mixture model to consider differences between item responses and response time patterns resulting from the solution behavior and rapid guessing behavior. The mixture model used both item response and response time information and considered multiple switch points for each examinee.

A variety of estimation methods have been proposed to estimate the parameters of the IRT and response time models. In the frequentist framework, the most common method is the marginal maximum likelihood estimation (MMLE) *via* expectation maximization algorithm (Bock and Aitkin, 1981; Baker and Kim, 2004). However, the main drawback of marginal maximum likelihood methods is the inevitable need for tedious approximation of the multidimensional integral using numerical integration (Bock and Schilling, 1997; Rabe-Hesketh et al., 2002, 2005) or Monte Carlo integration (Kuk, 1999; Skaug, 2002) when the latent variables are high dimensional. This is because the number of discrete quadrature points required increases exponentially as the number of latent variables increases linearly during the computation (Converse et al., 2021, p. 1465). Although the adaptive quadrature method has been used to deal with the computational deficiency by using a small number of quadrature points, the problem has not been completely solved (Jiang and Templin, 2019). In addition, the comparison method of the MMLE is simplistic; comparison methods other than the root mean square error of approximation are seldom used (Zhang et al., 2019). Compared with the MMLE method, first, the Bayesian method allows one to update knowledge by using proper informative priors based on previous studies, the posterior distribution being more precise than the likelihood or the prior alone (Jackman, 2009). The incorporation of proper informative priors into the Bayesian analysis can be used to obtain better results in the case of small to moderate sample sizes. In addition, even if weakly informative inaccurate priors are used, the performance of the Bayesian method does not deteriorate. Second, Bayesian estimation does not rely on asymptotic arguments and can give more reliable results for small samples (Lee and Song, 2004; Song and Lee, 2012). Third, another major advantage of Bayesian analysis is the ability to analyze models that are computationally heavy or impossible to estimate with MMLE. These include models with categorical outcomes with many latent variables and, thus, many dimensions of numerical integrations (Asparouhov and Muthén, 2010b; Muthén, 2010).

In the current study, an efficient Pólya–gamma Gibbs sampling algorithm (Polson et al., 2013) in a fully Bayesian framework is proposed to estimate the parameters of the mixture model of Wang and Xu (2015). Compared with traditional Bayesian sampling algorithms, e.g., the Metropolis–Hastings sampling algorithm (Metropolis et al., 1953; Hastings, 1970; Tierney, 1994; Chib and Greenberg, 1995; Chen et al., 2000), Gibbs sampling algorithm (Geman and Geman, 1984; Tanner and Wong, 1987; Gelfand and Smith, 1990; Albert, 1992; Béguin and Glas, 2001; Fox and Glas, 2001), and the advantages of the Pólya–gamma Gibbs sampling algorithm are presented from multiple perspectives. First, the Pólya–gamma Gibbs sampling algorithm avoids retrospective tuning in the Metropolis–Hastings sampling algorithm if we do not know how to choose a proper tuning parameter or if no value for the tuning parameter is appropriate. It always keeps the drawn samples accepted, thereby increasing the sampling efficiency (Zhang et al., 2020). Second, the Pólya–gamma Gibbs sampling algorithm can transform the non-conjugate model into the conjugate model by using augmented auxiliary variables. With the help of the traditional Gibbs sampling algorithm, posterior sampling is easier to implement (Polson et al., 2013). Third, in Bayesian estimation, prior distributions of model parameters and observed data likelihood produce a joint posterior distribution for the model parameters. The prior specifications and prior sensitivity are important aspects of Bayesian inference (Ghosh and A. Ghosh, 2000). In fact, the Pólya–gamma Gibbs sampling algorithm is not sensitive to the specification of the prior distribution. It can still obtain satisfactory results even if the proper or mis-specification priors are adopted (Zhang et al., 2020).

The rest of this article is organized as follows. Section 2 contains an introduction to the mixture hierarchical model and the corresponding identification restrictions. A detailed implementation of the Pólya–gamma Gibbs sampling algorithm is described in Section 3. In Section 4, two simulations focus on the parameter recovery performance of the Bayesian algorithm using the results of the model assessments. In addition, the quality of the Bayesian algorithm is investigated using high-state and large-scale computerized adaptive test data in Section 5. We conclude the article with a brief discussion in Section 6.

## 2. Models

Following Wang and Xu (2015), the mixture model is used to distinguish solution behavior from rapid guessing behavior. The correct response probability of examinee *i* on item *j* is assumed to follow a mixture decomposition


$$\begin{array}{l} P\left(Y_{ij}=1|\eta_{ij}\right)=\left(1-\eta_{ij}\right)P\left(Y_{ij}=1|\eta_{ij}=0\right)\\ \;+\eta_{ij}P\left(Y_{ij}=1|\eta_{ij}=1\right), \end{array}$$

where η_*ij*_ is a latent response behavior indicator variable, η_*ij*_ = 1 denotes the case where examinee *i* answers item *j* by rapid guessing behavior, and η_*ij*_ = 0 denotes the solution behavior. *P*(*Y*_*ij*_ = 1|η_*ij*_ = 0) quantifies the probability of a correct response resulting from the solution behavior, whereas *P*(*Y*_*ij*_ = 1|η_*ij*_ = 1) captures the probability of a correct response with the rapid guessing behavior. We use the two-parameter logistic (2PL; Birnbaum, 1968) model for the solution behavior,


$$\begin{array}{l} P\left(Y_{ij}=1|\eta_{ij}=0,\theta_{i},a_{j},b_{j}\right)=\frac{\exp\left[a_{j}\left(\theta_{i}-b_{j}\right)\right]}{1+\exp\left[a_{j}\left(\theta_{i}-b_{j}\right)\right]}, \end{array}$$

where *a*_*j*_ and *b*_*j*_ are the discrimination and difficulty parameters of item *j*, and θ_*i*_ denotes the ability of the *i*the examinee. The probability that examinee *i* answers item *j* correctly by the rapid guessing behavior is *g*_*j*_; this is an item-specific probability:


$$P\left(Y_{ij}=1|\eta_{ij}=1\right)=g_{j}.$$

In parallel with the mixture item response model, the observed response time $T_{ij}^{obs}$ is


$$T_{ij}^{obs}=\left(1-\eta_{ij}\right)T_{ij}+\eta_{ij}C_{ij},$$

where *T*_*ij*_ represents the time required for examinee *i* to respond to item *j* using solution behavior, and *C*_*ij*_ represents the time required for examinee *i* to respond to item *j* using rapid guessing behavior. Therefore, given latent indicator variable η_*ij*_, the density function of observed response time $T_{ij}^{obs}$ can be denoted as


$$p_{ij}\left(t_{ij}|\eta_{ij}\right)=\left(1-\eta_{ij}\right)f_{ij}\left(t_{ij}\right)+\eta_{ij}h_{ij}\left(t_{ij}\right),$$

where *f* and *h* represent corresponding density functions of *T*_*ijv*_ and *C*_*ijv*_.

Response times on test items have been modeled in various families of distributions in psychometric applications, including exponential (Scheiblechner, 1979), gamma (Maris, 1993), Weibull (Rouder et al., 2003), log-normal race (Rouder et al., 2015), and semi-parametric models (Wang et al., 2013). Response time data are non-negative, and their distributions tend to be positively skewed. The log transformation would move positively skewed distributions toward symmetric shapes. We chose the log-normal distribution (van der Linden, 2006) for response times with solution behavior:


$$\log\left(T_{ij}\right)=\lambda_{j}-\tau_{i}+e_{ij},\;e_{ij}\sim N\left(0,\sigma_{j}^{2}\right),$$

where λ_*j*_ is the time intensity of item *j*; a higher value of λ_*j*_ indicates that the item is expected to consume more time. τ_*i*_ is a speed parameter of examinee *i*; a higher value of τ_*i*_ means that the examinee works faster and a lower response time is expected. $\sigma_{j}^{2}$ allows for differences between the variances of log-times on different items. Following the “common-guessing” (Schnipke and Scrams, 1997), the response times of the guessing behavior have a common log-normal distribution


$$\log\left(C_{ij}\right)\sim N\left(\mu_{c},\sigma_{c}^{2}\right).$$

To capture across-person relationships between speed and accuracy, we assume that the ability and speed parameters have a bivariate normal distribution, to explore whether examinees with higher ability tend to answer items faster, i.e.,


$$\xi_{i}={\left(\theta_{i},\tau_{i}\right)}^{{}^{\prime }}\sim N\left(\mu_{P},\Sigma_{P}\right),$$

with mean vector


$$\mu_{P}={\left(\mu_{\theta },\mu_{\tau }\right)}^{{}^{\prime }}$$

and covariance matrix


$$\Sigma_{P}=\left(\begin{array}{cc} \sigma_{\theta }^{2} & \sigma_{\theta \tau }\\ \sigma_{\tau \theta } & \sigma_{\tau }^{2} \end{array}\right).$$

### 2.1. Model Identification

In the 2PL model, to eliminate the trade-off between ability θ and difficulty parameter *b* in location, we only need to fix the mean population level of ability to zero. That is, μ_θ_ = 0. To eliminate the trade-off between ability θ and discrimination parameter *a* in scale, we need to restrict the variance population level of ability to one. That is, $\sigma_{\theta }^{2}=1.$ For the response time model with the solution behavior, to eliminate the trade-off between speed parameter τ and time intensity parameter λ in location, we need to fix the mean population level of speed to zero. That is, μ_τ_ = 0.

There are several widely used identification restriction methods for two-parameter IRT models. The identification restrictions of our models are based on the following methods.

1. Fix the mean population level of ability to zero and the variance population level of ability to one (Lord and Novick, 1968; Bock and Aitkin, 1981; Fox and Glas, 2001; Fox, 2010). That is, θ~*N*(0, 1).
2. Restrict the sum of item difficulty parameters to zero and the product of item discrimination parameters to one (Fox and Glas, 2001; Fox, 2005, 2010). That is,
  $$\overset{J}{\underset{j=1}{\sum }}b_{j}=0\;\text{and }\overset{J}{\underset{j=1}{\prod }}a_{j}=1.$$
3. Fix the item difficulty parameter to a specific value, most often zero and restrict the discrimination parameter to a specific value, most often one (Fox and Glas, 2001; Fox, 2010). That is, *b*_1_ = 0 and *a*_1_ = 1.

## 3. Bayesian Estimation Using MCMC Sampling

Let $\Omega =(\eta_{ij},,\theta_{i},a_{j},b_{j},{\text{λ}}_{j},\tau_{i},\sigma_{j}^{2},\mu_{a},\sigma_{a}^{2},\mu_{b},\sigma_{b}^{2},\mu_{\text{λ}},\sigma_{\text{λ}}^{2},\mu_{c},\sigma_{c}^{2},g_{j},\sigma_{\theta \tau },\sigma_{\text{τ}}^{2},\pi_{i})$; then, the full joint posterior of person and item parameters given ***Y***, ***T***, and ***η*** is


(1) $$\begin{array}{l} L\left(\Omega |Y,T\right)\\ =\overset{N}{\underset{i=1}{\prod }}\overset{J}{\underset{j=1}{\prod }}{\left[\pi_{i}g_{j}h\left(t_{ij};\mu_{c},\sigma_{c}^{2}\right)\right]}^{\eta_{ij}.Y_{ij}}{\left[\pi_{i}\left(1-g_{j}\right)h\left(t_{ij};\mu_{c},\sigma_{c}^{2}\right)\right]}^{\eta_{ij}.\left(1-Y_{ij}\right)}\\ \times {\left[\left(1-\pi_{i}\right)P\left(Y_{ij}=1|\eta_{ij}=0,a_{j},b_{j},\theta_{i}\right)f\left(t_{ij};\lambda_{j},\tau_{i},\sigma_{j}^{2}\right)\right]}^{\left(1-\eta_{ij}\right).Y_{ij}}\\ \times {\left[\left(1-\pi_{i}\right)P\left(Y_{ij}=0|\eta_{ij}=0,a_{j},b_{j},\theta_{i}\right)f\left(t_{ij};\lambda_{j},\tau_{i},\sigma_{j}^{2}\right)\right]}^{\left(1-\eta_{ij}\right).\left(1-Y_{ij}\right)}\\ \times p\left(\theta_{i},\tau_{i};\mu_{p},\Sigma_{p}\right)p\left(a_{j}\right)p\left(b_{j}\right)p\left(\lambda_{j}\right)p\left(\mu_{p},\Sigma_{p}\right), \end{array}$$

where π_*i*_ is the probability that examinee *i* uses the rapid guessing behavior, i.e., π_*i*_ = *P*(η_*ij*_ = 1).

### 3.1. Pólya–Gamma Gibbs Sampling Algorithm

Polson et al. (2013) proposed a new data augmentation strategy for fully Bayesian inference in logistic regression. This data augmentation approach used a new class of Pólya–gamma distribution, in contrast to the data augmentation algorithm of Albert and Chib (1993), which was based on a truncated normal distribution. Here, we introduce the Pólya–gamma distribution.

**Definition**: Let ${\left\{B_{k}\right\}}_{k=1}^{+\infty }$ be an *independent and identically distributed* random variable sequence from a gamma distribution with parameters β and 1. That is, *B*_*k*_~gamma(β, 1). A random variable *W* follows a Pólya–gamma distribution with parameters β > 0 and ϱ ∈ *R*, denoted *W* ~ PG(β, ϱ), if


$$W\overset{D}{=}\frac{1}{2\pi }\overset{+\infty }{\underset{k=1}{\sum }}\frac{B_{k}}{{\left(k-\frac{1}{2}\right)}^{2}+\frac{\varrho^{2}}{4\pi^{2}}},$$

where $\overset{D}{=}$ denotes equality in distribution. In fact, the Pólya–gamma distribution is an infinite mixture of gamma distributions, which provides the ability to sample from gamma distributions.

Based on Theorem 1 of Polson et al. (2013, page 1341, Equation 7), the likelihood contribution of the *i*th examinee answering the *j*th item under the solution behavior category η_*ij*_ = 0 can be expressed as


(2) $$\begin{array}{l} L\left(a_{j},b_{j},\theta_{i}\right)=\frac{{\left\{\exp\left[a_{j}\left(\theta_{i}-b_{j}\right)\right]\right\}}^{Y_{ij}}}{1+\left\{\exp\left[a_{j}\left(\theta_{i}-b_{j}\right)\right]\right\}}\propto \exp\left\{k_{ij}\left[a_{j}\left(\theta_{i}-b_{j}\right)\right]\right\}\\ \;\times \overset{\infty }{\underset{0}{\int }}\exp\left\{-\frac{W_{ij}{\left[a_{j}\left(\theta_{i}-b_{j}\right)\right]}^{2}}{2}\right\}p\left(W_{ij}|1,0\right)dW_{ij}, \end{array}$$

where $k_{ij}=Y_{ij}-\frac{1}{2}.$ *p*(*W*_*ij*_|1, 0) is the conditional density of *W*_*ij*_. That is, *W*_*ij*_ ~ PG(1, 0). The auxiliary variable *W*_*ij*_ follows a Pólya–gamma distribution with parameters (1, 0). Within the solution behavior category η_*ij*_ = 0, the full conditional distribution of ***a***, ***b***, ***θ*** given the auxiliary variables, ***W*** can be written as


(3) $$\begin{array}{l} p\left(a,b,\theta |\eta ,W,Y\right)\\ \propto {\left\{\overset{N}{\underset{i=1}{\prod }}\overset{J}{\underset{j=1}{\prod }}\left[\exp\left\{k_{ij}\left[a_{j}\left(\theta_{i}-b_{j}\right)\right]\right\}\exp\left[-\frac{W_{ij}{\left[a_{j}\left(\theta_{i}-b_{j}\right)\right]}^{2}}{2}\right]\right]\right\}}^{\text{I}\left(\eta_{ij}=0\right)}\\ \times {\left\{\overset{N}{\underset{i=1}{\prod }}p\left(\theta_{i}|\tau_{i},\mu_{P},\Sigma_{P}\right)\right\}}^{\text{I}\left(\eta_{ij}=0\right)}{\left\{\overset{J}{\underset{j=1}{\prod }}\left[p\left(a_{j}\right)p\left(b_{j}\right)\right]\right\}}^{\text{I}\left(\eta_{ij}=0\right)}, \end{array}$$

where *p*(*a*_*j*_) and *p*(*b*_*j*_) are the prior distributions for *a*_*j*_ and *b*_*j*_. It is known that there are relationships between the latent ability and speed parameter, which can be constructed by a bivariate normal prior distribution $\left(\begin{array}{c} \theta_{i}\\ \tau_{i} \end{array}\right)\sim N\left(\left(\begin{array}{c} \mu_{\theta }\\ \mu_{\tau } \end{array}\right),\Sigma_{P}\right)$. Therefore, the conditional prior distribution of θ_*i*_ is the normal distribution


$$\theta_{i}|\tau_{i},\mu_{P},\Sigma_{P}\sim N\left(\mu_{\theta |\tau },\sigma_{\theta |\tau }^{2}\right),$$

where $\mu_{\theta |\tau }=\mu_{\theta }+\sigma_{\theta \tau }\sigma_{\tau }^{-2}\left(\tau_{i}-\mu_{\tau }\right)$ and $\sigma_{\theta |\tau }^{2}=\sigma_{\theta }^{2}-\sigma_{\theta \tau }\sigma_{\tau }^{-2}\sigma_{\tau \theta }.$

**Step 1**: Sample the auxiliary variable *W*_*ij*_, within the solution behavior category η_*ij*_ = 0, given the item discrimination and difficulty parameters *a*_*j*_, *b*_*j*_ and the ability θ_*i*_. According to Equation (1), the full conditional posterior distribution of the random auxiliary variable *W*_*ij*_ is given by


$$f\left(W_{ij}|a_{j},b_{j},\theta_{i}\right)\propto \exp\left[-\frac{W_{ij}{\left[a_{j}\left(\theta_{i}-b_{j}\right)\right]}^{2}}{2}\right]p\left(W_{ij}|1,0\right).$$

According to Biane et al. (2001) and Polson et al. (2013; p. 1341), the density function *p*(*W*_*ij*_|1, 0) can be written as


$$p\left(W_{ij}|1,0\right)=\overset{\infty }{\underset{v=0}{\sum }}{\left(-1\right)}^{v}\frac{\left(2k+1\right)}{\sqrt{2\pi W_{ij}}}\exp\left[-\frac{{\left(2k+1\right)}^{2}}{8W_{ij}}\right].$$

Therefore, *f*(*W*_*ij*_|*a*_*j*_, *b*_*j*_, θ_*i*_) is proportional to


$$\overset{\infty }{\underset{v=0}{\sum }}{\left(-1\right)}^{v}\frac{\left(2k+1\right)}{\sqrt{2\pi W_{ij}}}\exp\left[-\frac{{\left(2k+1\right)}^{2}}{8W_{ij}}-\frac{W_{ij}{\left[a_{j}\left(\theta_{i}-b_{j}\right)\right]}^{2}}{2}\right].$$

Finally, the specific form of the full conditional distribution of *W*_*ij*_ is as follows:


$$W_{ij}\sim \text{PG}\left(1,|a_{j}\left(\theta_{i}-b_{j}\right)|\right).$$

Next, Gibbs samplers are used to draw the item parameters.

**Step 2**: Sample the discrimination parameter *a*_*j*_ for each item *j*. The prior distribution of *a*_*j*_ is assumed to follow a truncated normal distribution, i.e., $a_{j}\sim N\left(\mu_{a},\sigma_{a}^{2}\right)\text{I}\left(a_{j}>0\right)$. Given ***Y***, ***W***, *b*_*j*_, and ***θ***, the fully conditional posterior distribution of *a*_*j*_ is given by


$$\begin{array}{l} p\left(a_{j}|Y,W,b_{j},\theta \right)\\ \;\propto \overset{N}{\underset{i=1}{\prod }}\left\{\frac{{\left\{\exp\left[a_{j}\left(\theta_{i}-b_{j}\right)\right]\right\}}^{Y_{ij}}}{1+\exp\left[a_{j}\left(\theta_{i}-b_{j}\right)\right]}f\left(W_{ij}|a_{j},b_{j},\theta_{i}\right)\right\}p\left(a_{j}\right),\; \end{array}$$

where *f*(*W*_*ij*_|*a*_*j*_, *b*_*j*_, θ_*i*_) is given by the following equation (for details, refer to Polson et al., 2013; p. 1341):


$$\begin{array}{l} f\left(W_{ij}|a_{j},b_{j},\theta_{i}\right)=\left\{\cosh\left(2^{-1}|a_{j}\left(\theta_{i}-b_{j}\right)|\right)\right\}\frac{2^{0}}{\Gamma \left(1\right)}\\ \;\times \overset{\infty }{\underset{v=0}{\sum }}{\left(-1\right)}^{v}\frac{\left(2k+1\right)}{\sqrt{2\pi W_{ij}}}\\ \;\times \exp\left[-\frac{{\left(2k+1\right)}^{2}}{8W_{ij}}-\frac{W_{ij}{\left[a_{j}\left(\theta_{i}-b_{j}\right)\right]}^{2}}{2}\right]. \end{array}$$

After rearrangement, the full conditional posterior distribution of *a*_*j*_ can be written as follows:


$$\begin{array}{l} p\left(a_{j}|Y,W,b_{j},\theta \right)\propto \overset{N}{\underset{i=1}{\prod }}\{\frac{{\left\{\exp\left[a_{j}\left(\theta_{i}-b_{j}\right)\right]\right\}}^{Y_{ij}}}{1+\exp\left[a_{j}\left(\theta_{i}-b_{j}\right)\right]}\\ \;\times \left[\cosh\left(2^{-1}\left|a_{j}\left(\theta_{i}-b_{j}\right)\right|\right)\right]\\ \;\times \exp\left[-\frac{{\left[a_{j}\left(\theta_{i}-b_{j}\right)\right]}^{2}W_{ij}}{2}\right]\}p\left(a_{j}\right). \end{array}$$

Therefore, the fully conditional posterior distribution of *a*_*j*_ follows a normal distribution truncated at 0 with mean


$$\text{Va}{\text{r}}_{a_{j}}\times \left(\mu_{a}\sigma_{a}^{-2}+\left[\overset{N}{\underset{i=1}{\sum }}W_{ij}{\left(\theta_{i}-b_{j}\right)}^{2}\right]\left\{\frac{\left[\overset{N}{\underset{i=1}{\sum }}\left(1-2Y_{ij}\right)\left(\theta_{i}-b_{j}\right)\right]}{2\left[\overset{N}{\underset{i=1}{\sum }}W_{ij}{\left(\theta_{i}-b_{j}\right)}^{2}\right]}\right\}\right)$$

and variance


$$\text{Va}{\text{r}}_{a_{j}}={\left\{\sigma_{a}^{-2}+\left[\overset{N}{\underset{i=1}{\sum }}W_{ij}{\left(\theta_{i}-b_{j}\right)}^{2}\right]\right\}}^{-1}.$$

**Step 3**: Sample the difficulty parameter *b*_*j*_ for each item *j*. The prior distribution of *b*_*j*_ is assumed to follow a normal distribution with mean μ_*b*_ and $\sigma_{b}^{2}.$ That is, $b_{j}\sim N\left(\mu_{b},\sigma_{b}^{2}\right).$ Similarly, given ***Y***, ***W***, *a*_*j*_, and ***θ***, the fully conditional posterior distribution of *b*_*j*_ is given by


$$\begin{array}{l} p\left(b_{j}|Y,W,a_{j},\theta \right)\propto \overset{N}{\underset{i=1}{\prod }}\{\frac{{\left\{\exp\left[a_{j}\left(\theta_{i}-b_{j}\right)\right]\right\}}^{Y_{ij}}}{1+\exp\left[a_{j}\left(\theta_{i}-b_{j}\right)\right]}\\ \;\times \left[\cosh\left(2^{-1}\left|a_{j}\left(\theta_{i}-b_{j}\right)\right|\right)\right]\\ \;\times \exp\left[-\frac{{\left[a_{j}\left(\theta_{i}-b_{j}\right)\right]}^{2}W_{ij}}{2}\right]\}p\left(b_{j}|\mu_{b},\sigma_{b}^{2}\right). \end{array}$$

Therefore, the fully conditional posterior distribution of *b*_*j*_ follows a normal distribution with mean


$$\text{Va}{\text{r}}_{b_{j}}\times \left(\mu_{b}\sigma_{b}^{-2}+\overset{N}{\underset{i=1}{\sum }}\left[a_{j}^{2}W_{ij}\right]\left(\frac{\overset{N}{\underset{i=1}{\sum }}\left(2a_{j}^{2}\theta_{i}W_{ij}-2Y_{ij}a_{j}+a_{j}\right)}{2\overset{N}{\underset{i=1}{\sum }}\left[a_{j}^{2}W_{ij}\right]}\right)\right)$$

and variance


$$\text{Va}{\text{r}}_{b_{j}}={\left\{\sigma_{b}^{-2}+\overset{N}{\underset{i=1}{\sum }}\left[a_{j}^{2}W_{ij}\right]\right\}}^{-1}$$

**Step 4**: Sample the ability parameter θ_*i*_ for each examinee *i*. The conditional prior distribution of θ_*i*_ is assumed to follow a normal distribution with mean $\mu_{\theta |\tau }=\mu_{\theta }+\sigma_{\theta \tau }\sigma_{\tau }^{-2}\left(\tau_{i}-\mu_{\tau }\right)$ and $\sigma_{\theta |\tau }^{2}=\sigma_{\theta }^{2}-\sigma_{\theta \tau }\sigma_{\tau }^{-2}\sigma_{\tau \theta }.$ That is, $\theta_{i}\sim N\left(\mu_{\theta |\tau },\sigma_{\theta |\tau }^{2}\right).$ Given ***Y***, ***W***, ***a*** and ***b***, the fully conditional posterior distribution of θ_*i*_ is given by


$$\begin{array}{l} \;p\left(\theta_{i}|Y,W,a,b\right)\\ \propto \overset{J}{\underset{j=1}{\prod }}\{\frac{{\left\{\exp\left[a_{j}\left(\theta_{i}-b_{j}\right)\right]\right\}}^{Y_{ij}}}{1+\exp\left[a_{j}\left(\theta_{i}-b_{j}\right)\right]}\left[\cosh\left(2^{-1}\left|a_{j}\left(\theta_{i}-b_{j}\right)\right|\right)\right]\\ \times \exp\left[-\frac{{\left[a_{j}\left(\theta_{i}-b_{j}\right)\right]}^{2}W_{ij}}{2}\right]\}p\left(\theta_{i}|\mu_{\theta |\text{τ}},\sigma_{\theta |\text{τ}}^{2}\right). \end{array}$$

Therefore, the fully conditional posterior distribution of θ_*i*_ follows a normal distribution with mean


$$\text{Va}{\text{r}}_{\theta_{i}}\times \left(\mu_{\theta |\tau }\sigma_{\theta |\tau }^{-2}+\overset{J}{\underset{j=1}{\sum }}\left[a_{j}^{2}W_{ij}\right]\left(\frac{\overset{J}{\underset{j=1}{\sum }}\left(2Y_{ij}a_{j}+2a_{j}^{2}b_{j}W_{ij}-a_{j}\right)}{2\overset{J}{\underset{j=1}{\sum }}\left[a_{j}^{2}W_{ij}\right]}\right)\right)$$

and variance


$$\text{Va}{\text{r}}_{\theta_{i}}={\left\{\sigma_{\theta |\tau }^{-2}+\overset{J}{\underset{j=1}{\sum }}\left[a_{j}^{2}W_{ij}\right]\right\}}^{-1}.$$

**Step 5**: Sample the response behavior variable η_*ij*_. The fully conditional posterior distribution of η_*ij*_ is a Bernoulli distribution with success probability


$$\begin{array}{ll} \;\frac{\pi_{i}g_{j}h\left(T_{ij};\mu_{c},\sigma_{c}^{2}\right)}{\pi_{i}g_{j}h\left(T_{ij};\mu_{c},\sigma_{c}^{2}\right)+\left(1-\pi_{i}\right)P\left(Y_{ij}=1|\theta_{i},a_{j},b_{j}\right)f\left(T_{ij};\lambda_{j},\tau_{i},\sigma_{j}^{2}\right)}, & \text{if }Y_{ij}=1,\\ \frac{\pi_{i}\left(1-g_{j}\right)h\left(T_{ij};\mu_{c},\sigma_{c}^{2}\right)}{\pi_{i}\left(1-g_{j}\right)h\left(T_{ij};\mu_{c},\sigma_{c}^{2}\right)+\left(1-\pi_{i}\right)P\left(Y_{ij}=0|\theta_{i},a_{j},b_{j}\right)f\left(T_{ij};\lambda_{j},\tau_{i},\sigma_{j}^{2}\right)}, & \text{if }Y_{ij}=0. \end{array}$$

**Step 6**: Sample π_*i*_. Given a *Beta*(ι_1_, ι_2_) prior and $\overset{J}{\underset{j=1}{\sum }}\eta_{ij}\sim Binomial\left(J,\pi_{i}\right)$, the fully conditional posterior of π_*i*_ is


$$\pi_{i}\sim Beta\left(\iota_{1}+\overset{J}{\underset{j=1}{\sum }}\eta_{ij},\;\iota_{2}+J-\overset{J}{\underset{j=1}{\sum }}\eta_{ij}\right).$$

**Step 7**: Sample *g*_*j*_. Given a *Beta*(ι_3_, ι_4_) prior, within the guessing behavior category η_*ij*_ = 1, the total number of people engaging in rapid guessing behavior on item *j* is $\overset{N}{\underset{i=1}{\sum }}\eta_{ij}$, and the number of correct items is $\overset{N}{\underset{i=1}{\sum }}\eta_{ij}Y_{ij}$; thus, $\overset{N}{\underset{i=1}{\sum }}\eta_{ij}Y_{ij}\sim Binomial\left(\overset{N}{\underset{i=1}{\sum }}\eta_{ij},g_{j}\right)$. The fully conditional posterior is


$$g_{j}\sim Beta\left(\iota_{3}+\overset{N}{\underset{i=1}{\sum }}\eta_{ij}Y_{ij},\;\iota_{4}+\overset{N}{\underset{i=1}{\sum }}\eta_{ij}-\overset{N}{\underset{i=1}{\sum }}\eta_{ij}Y_{ij}\right).$$

**Step 8**: Sample τ_*i*_. The conditional prior distribution of τ_*i*_ is assumed to follow a normal distribution with mean $\mu_{\tau |\theta }=\mu_{\tau }+\sigma_{\tau \theta }\sigma_{\theta }^{-2}\left(\theta_{i}-\mu_{\theta }\right)$ and $\sigma_{\tau |\theta }^{2}=\sigma_{\tau }^{2}-\sigma_{\tau \theta }\sigma_{\theta }^{-2}\sigma_{\theta \tau }.$ That is, $\tau_{i}\sim N\left(\mu_{\tau |\theta },\sigma_{\tau |\theta }^{2}\right).$ The fully conditional posterior distribution of τ_*i*_ given ***T***^*obs*^, ***θ***, ***λ*** , $\sigma_{j}^{2},$ ***μ***_*P*_, ***Σ***_*P*_, ***η*** is proportional to


$$\overset{J}{\underset{j=1}{\prod }}f{\left(t_{ij};\lambda_{jv},\tau_{i},\sigma_{j}^{2}\right)}^{\left(1-\eta_{ij}\right)}p\left(\tau_{i}|\mu_{\theta |\tau },\sigma_{\theta |\tau }^{2}\right).$$

The fully conditional posterior distribution of τ_*i*_ is


$$N\left(\sigma_{\tau_{i}^{*}}^{2}\left(\frac{\sigma_{\theta \tau }\theta_{i}}{\sigma_{\tau }^{2}-\sigma_{\theta \tau }^{2}}+\overset{J}{\underset{j=1}{\sum }}\left[\left(1-\eta_{ij}\right)\sigma_{j}^{-2}\left(\lambda_{j}-\log t_{ij}\right)\right]\right),\;\sigma_{\tau_{i}^{*}}^{2}\right),$$

where $\sigma_{\tau_{i}^{*}}^{2}={\left({\left(\sigma_{\tau }^{2}-\sigma_{\theta \tau }^{2}\right)}^{-1}+\sum_{j=1}^{J}\left[\left(1-\eta_{ij}\right)\sigma_{j}^{-2}\right]\right)}^{-1}$.

**Step 9**: Sample λ_*j*_. The fully conditional posterior distribution of the intensity parameter given the parameters ***T***^*obs*^, ***τ***, $\sigma_{j}^{2},$ ***μ***_*I*_, ***Σ***_*I*_, ***η*** is


$$\begin{array}{l} \;p\left(\lambda_{j}|T_{j}^{obs},\tau ,\sigma_{j}^{2},\mu_{\lambda },\sigma_{\lambda }^{2},\eta \right)\\ \propto \overset{N}{\underset{i=1}{\prod }}f{\left(t_{ij};\lambda_{j},\tau_{i},\sigma_{j}^{2}\right)}^{\left(1-\eta_{ij}\right)}p\left(\lambda_{j}|\mu_{\lambda },\sigma_{\lambda }^{2}\right), \end{array}$$

where $\lambda_{j}\sim N\left(\mu_{\lambda },\sigma_{\lambda }^{2}\right).$ The fully conditional posterior distribution of λ_*j*_ is


$$N\left(\sigma_{\lambda_{j}^{*}}^{2}\left(\mu_{\lambda }\sigma_{\lambda }^{-2}+\overset{N}{\underset{i=1}{\sum }}\left(1-\eta_{ij}\right)\left(\log t_{ij}+\tau_{i}\right)\sigma_{j}^{-2}\right),\sigma_{\lambda_{j}^{*}}^{2}\right),$$

where $\sigma_{{\text{λ}}_{j}^{*}}^{2}={\left(\sigma_{\text{λ}}^{-2}+\sigma_{j}^{-2}\sum_{i=1}^{N}\left(1-\eta_{ij}\right)\right)}^{-1}$.

**Step 10**: Sample $\sigma_{j}^{2}$. A prior for $\sigma_{j}^{2}$ is an inverse-gamma distribution, *IG*(υ_1_, ω_1_). The fully conditional posterior distribution of $\sigma_{j}^{2}$ is


$$IG\left(\upsilon_{1}+\frac{\overset{N}{\underset{i=1}{\sum }}\left(1-\eta_{ij}\right)}{2},\omega_{1}+\frac{\overset{N}{\underset{i=1}{\sum }}\left[\left(1-\eta_{ij}\right){\left(\log t_{ij}-\lambda_{j}+\tau_{i}\right)}^{2}\right]}{2}\right).$$

**Step 11**: Sample μ_*c*_. We assume a uniform prior *p*(μ_*c*_)∝1. The fully conditional posterior distribution of μ_*c*_ is proportional to


$$p\left(\mu_{c}|T,\eta \right)\propto \overset{N}{\underset{i=1}{\prod }}\overset{J}{\underset{j=1}{\prod }}f{\left(t_{ij};\mu_{c},\sigma_{c}^{2}\right)}^{\eta_{ij}}p\left(\mu_{c}\right).$$

The fully conditional posterior distribution of μ_*c*_ is


$$\begin{array}{l} \mu_{c}|T,\eta \sim N({\left(\sum_{i=1}^{N}\sum_{j=1}^{J}\eta_{ij}\right)}^{-1}\left(\sum_{i=1}^{N}\sum_{j=1}^{J}\eta_{ij}\log t_{ij}\right),\\ \;{\left(\sum_{i=1}^{N}\sum_{j=1}^{J}\eta_{ij}\right)}^{-1}\sigma_{c}^{2}). \end{array}$$

**Step 12**: Sample $\sigma_{c}^{2}$. We assume that the variance parameter follows an inverse-gamma prior distribution, *IG*(υ_2_, ω_2_). The fully conditional posterior distribution of $\sigma_{c}^{2}$ given ***T***, μ_*c*_, υ_2_, ω_2_, ***η*** is proportional to


$$\begin{array}{l} p\left(\sigma_{c}^{2}|T,\mu_{c},\upsilon_{2},\omega_{2},\eta \right)\propto \overset{N}{\underset{i=1}{\prod }}\overset{J}{\underset{j=1}{\prod }}f{\left(t_{ij};\mu_{c},\sigma_{c}^{2}\right)}^{\eta_{ij}}p\left(\sigma_{c}^{2}\right). \end{array}$$

The fully conditional posterior distribution of $\sigma_{c}^{2}$ is


$$\begin{array}{l} \sigma_{c}^{2}|T,\mu_{c},\upsilon_{2},\omega_{2},\eta \\ \sim IG\left(\upsilon_{1}+\frac{\overset{N}{\underset{i=1}{\sum }}\overset{J}{\underset{j=1}{\sum }}\eta_{ij}}{2},\omega_{1}+\frac{\overset{N}{\underset{i=1}{\sum }}\overset{J}{\underset{j=1}{\sum }}\eta_{ij}{\left(\log t_{ij}-\mu_{c}\right)}^{2}}{2}\right). \end{array}$$

### 3.2. Metropolis–Hastings Sampling Algorithm

In order to estimate the constrained covariance matrix $\Sigma_{P}=\left(\begin{array}{cc} 1\; & \sigma_{\theta \tau }\\ \sigma_{\tau \theta }\; & \sigma_{\tau }^{2} \end{array}\right)$(where $\sigma_{\theta }^{2}$ is restricted to be equal to 1 owing to the model identification limitation), we need to update each element of the constrained covariance matrix separately using the Metropolis–Hastings algorithm.

**Step 13**: Sample the correlation σ_θτ_ between ***θ*** and ***τ***. The identification constraints induce a restricted covariance matrix. The new value $\sigma_{\theta \tau }^{*}$ is sampled from a truncated normal distribution $N\left(\sigma_{\theta \tau }^{\left(r-1\right)},s_{01}^{2}\right)I\left(-p_{01}<\sigma_{\theta \tau }^{*}<p_{01}\right)$, where $p_{01}=\sqrt{\sigma_{\tau }^{2,\left(r-1\right)}}$. Therefore, the probability of acceptance $\alpha \left(\sigma_{\theta \tau }^{\left(r-1\right)},\sigma_{\theta \tau }^{*}\right)$ can be written as


$$\min\left\{1,\frac{\overset{N}{\underset{i=1}{\prod }}p\left(\tau_{i}|\theta_{i}^{{}^{\left(r\right)}},\sigma_{\tau }^{2,\left(r-1\right)},\sigma_{\theta \tau }^{*}\right)p\left(\sigma_{\theta \tau }^{*}\right)\left(\Phi \left(\frac{p_{01}-\sigma_{\theta \tau }^{\left(r-1\right)}}{s_{01}}\right)-\Phi \left(\frac{-p_{01}-\sigma_{\theta \tau }^{\left(r-1\right)}}{s_{01}}\right)\right)}{\overset{N}{\underset{i=1}{\prod }}p\left(\tau_{i}|\theta_{i}^{{}^{\left(r\right)}},\sigma_{\tau }^{2,\left(r-1\right)},\sigma_{\theta \tau }^{\left(r-1\right)}\right)p\left(\sigma_{\theta \tau }^{\left(r-1\right)}\right)\left(\Phi \left(\frac{p_{01}-\sigma_{\theta \tau }^{*}}{s_{01}}\right)-\Phi \left(\frac{-p_{01}-\sigma_{\theta \tau }^{*}}{s_{01}}\right)\right)}\right\};$$

otherwise, $\sigma_{\theta \tau }^{\left(r-1\right)}=\sigma_{\theta \tau }^{*}$, where *p*(τ_*i*_|θ_*i*_) is the conditional density function of the speed parameter, $s_{01}^{2}$ is the proposal variance, and *p*(σ_θτ_) is the density of the uniform prior.

**Step 14**: Sample $\sigma_{\tau }^{2}$. The new value $\sigma_{\tau }^{2,*}$ is sampled from a truncated normal distribution $N\left(\sigma_{\tau }^{2,\left(r-1\right)},s_{02}^{2}\right)I\left(\sigma_{\tau }^{2,*}>{\left(\sigma_{\theta^{\left(2\right)}\tau }^{*}\right)}^{2}=p_{0}\right)$. Therefore, the probability of acceptance $\alpha \left(\sigma_{\tau }^{2,\left(r-1\right)},\sigma_{\tau }^{2,*}\right)$ can be written as


$$\min\left\{1,\frac{\overset{N}{\underset{i=1}{\prod }}p\left(\tau_{i}|\theta_{i}^{{}^{\left(r\right)}},\sigma_{\tau }^{2,*},\sigma_{\theta \tau }^{\left(r\right)}\right)p\left(\sigma_{\tau }^{2,*};\kappa ,\vartheta \right)\left(1-\Phi \left(\frac{p_{0}-\sigma_{\tau }^{2,\left(r-1\right)}}{s_{02}}\right)\right)}{\overset{N}{\underset{i=1}{\prod }}p\left(\tau_{i}|\theta_{i}^{{}^{\left(r\right)}},\sigma_{\tau }^{2,\left(r-1\right)},\sigma_{\theta \tau }^{\left(r\right)}\right)p\left(\sigma_{\tau }^{2,\left(r-1\right)};\kappa ,\vartheta \right)\left(1-\Phi \left(\frac{p_{0}-\sigma_{\tau }^{2,*}}{s_{02}}\right)\right)}\right\};$$

otherwise, $\sigma_{\tau }^{2,*}=\sigma_{\tau }^{2,\left(r-1\right)}$, where $s_{02}^{2}$ is the proposal variance, and $p\left(\sigma_{\tau }^{2};\kappa ,\vartheta \right)$ is the density function of the scaled inverse chi-squared distribution with degrees of freedom and the scale parameter.

### 3.3. Bayesian Model Assessment

Two Bayesian model assessment methods were developed to evaluate the fit of the two models. The new model is a mixture model that combines responses and response times to detect rapid guessing behavior. The other model does not consider the mixture structure. Spiegelhalter et al. (2002) proposed the deviance information criterion (DIC) as a way to evaluate model fit based on Bayesian posterior estimates by considering the trade-off relationship between the adequacy of the model fitting and the number of model parameters. Write ***Λ* =**(***Λ***_*ij*_, *i* = 1, ..., *N*. *j* = 1, ..., *J*.), where $\Lambda_{ij}={\left(\eta_{ij},\theta_{i},a_{j},b_{j},\lambda_{j},\tau_{i},\sigma_{j}^{2},\mu_{c},\sigma_{c}^{2},g_{j},\pi_{i}\right)}^{{}^{\prime }}.$ Let {***Λ***^(1)^, ..., ***Λ***^(*M*)^}, where $\Lambda^{\left(m\right)}={\left(\eta_{ij}^{\left(m\right)},\theta_{i}^{\left(m\right)},a_{j}^{\left(m\right)},b_{j}^{\left(m\right)},\lambda_{j}^{\left(m\right)},\tau_{i}^{\left(m\right)},\sigma_{j}^{2,\left(m\right)},\mu_{c}^{\left(m\right)},\sigma_{c}^{2,\left(m\right)},g_{j}^{\left(m\right)},\pi_{i}^{\left(m\right)}\right)}^{{}^{\prime }}$ for *m* = 1, ..., *M*, denotes an Markov chain Monte Carlo (MCMC) sample from the posterior distribution in (1). The logarithm of the joint likelihood function evaluated at ***Λ***^(*m*)^ is given by


(4) $$\begin{array}{l} \log f\left(Y,T|\Lambda^{\left(m\right)}\right)=\overset{N}{\underset{i=1}{\sum }}\overset{J}{\underset{j=1}{\sum }}\log f\left(Y_{ij},T_{ij}|\Lambda_{ij}^{\left(m\right)}\right), \end{array}$$

where


$$\begin{array}{l} f\left(Y_{ij},T_{ij}|\Lambda_{ij}\right)\\ ={\left[\pi_{i}g_{j}h\left(T_{ij}|\mu_{c},\sigma_{c}^{2}\right)\right]}^{\eta_{ij}.Y_{ij}}{\left[\pi_{i}\left(1-g_{j}\right)h\left(T_{ij}|\mu_{c},\sigma_{c}^{2}\right)\right]}^{\eta_{ij}.\left(1-Y_{ij}\right)}\\ \times {\left[\left(1-\pi_{i}\right)P\left(Y_{ij}=1|\eta_{ij}=0\right)f\left(T_{ij}|\lambda_{j},\tau_{i},\sigma_{j}^{2}\right)\right]}^{\left(1-\eta_{ij}\right).Y_{ij}}\\ \times {\left[\left(1-\pi_{i}\right)P\left(Y_{ij}=0|\eta_{ij}=0\right)f\left(T_{ij}|\lambda_{j},\tau_{i},\sigma_{j}^{2}\right)\right]}^{\left(1-\eta_{ij}\right).\left(1-Y_{ij}\right)}. \end{array}$$

As the log-likelihood function $\log f\left(Y_{ij},T_{ij}|\Lambda_{ij}^{\left(r\right)}\right),$ *i* = 1, ..., *N*. *j* = 1, ..., *J*, is readily available from the R outputs, log*f*(***Y***, ***T***|***Λ***^(*r*)^) in (4) is easy to compute. The DIC can be calculated as follows:


(5) $$\text{DIC=}\hat{\text{Dev}(\Lambda )}+2P_{D}=\hat{\text{Dev}(\Lambda )}+2\left[\bar{\text{Dev}(\Lambda )}-\hat{\text{Dev}(\Lambda )}\right],$$

where


$$\begin{array}{l} \bar{\text{Dev}\left(\Lambda \right)}=-\frac{2}{M}\overset{M}{\underset{m=1}{\sum }}\log f\left(Y,T|\Lambda^{\left(m\right)}\right)\text{and}\\ \hat{\text{Dev}\left(\Lambda \right)}=-2\underset{1\leq m\leq M}{\max}\log f\left(Y,T|\Lambda^{\left(m\right)}\right). \end{array}$$

In (5), $\bar{\text{Dev}\left(\Lambda \right)}$ is a Monte Carlo estimate of the posterior expectation of the deviance function Dev(***Λ***) = −2log*f*(***Y***, ***T***|***Λ***). $\hat{\text{Dev}\left(\Lambda \right)}$ is an approximation of $\text{Dev}\left(\hat{\Lambda }\right),$ where $\hat{\Lambda }$ is the posterior mode, when the prior is relatively non-informative, and $P_{D}=\bar{\text{Dev}\left(\Lambda \right)}-\hat{\text{Dev}\left(\Lambda \right)}$ is the effective number of parameters. The model with a smaller DIC value fits the data better.

Another method to compare the fit of the two models is to use the logarithm of the pseudomarginal likelihood (LPML; Geisser and Eddy, 1979; Ibrahim et al., 2001) by calculating the conditional predictive ordinates (CPO) index. Next, the formulas for computing the CPO and LPML are given. Letting $U_{ij,\max}=\underset{1\leq m\leq M}{\max}\left\{-\log f\left(Y_{ij},T_{ij}|\Lambda_{ij}^{\left(m\right)}\right)\right\},$ a Monte Carlo estimate of the CPO (Gelfand et al., 1992; Chen et al., 2000) is given by


(6) $$\begin{array}{l} \log\hat{\left(\text{CP}{\text{O}}_{ij}\right)}=-U_{ij,\max}\\ -\log\left[\frac{1}{M}\overset{M}{\underset{m=1}{\sum }}\exp\left\{-\log f\left(Y_{ij},T_{ij}|\Lambda_{ij}^{\left(m\right)}\right)-U_{ij,\max}\right\}\right]. \end{array}$$

Note that the maximum value adjustment used in $\log\hat{\left(\text{CP}{\text{O}}_{ij}\right)}$ plays an important part in numerical stabilization when computing $\exp\left\{-\log f\left(Y_{ij},T_{ij}|\Lambda_{ij}^{\left(m\right)}\right)-U_{ij,\max}\right\}$ in (6). A summary statistic of the $\hat{\text{CP}{\text{O}}_{ij}}$ is the sum of their logarithms, which is called the LPML and given by


$$\text{LPML}=\overset{N}{\underset{i=1}{\sum }}\overset{J}{\underset{j=1}{\sum }}\log\hat{\left(\text{CP}{\text{O}}_{ij}\right)}.$$

A model with a larger LPML has a better fit to the data.

## 4. Simulation Studies

### 4.1. Simulation 1

This simulation study was conducted to evaluate the recovery performance of the Pólya–gamma Gibbs sampling algorithm under different simulation conditions.

**Simulation Designs**

The following conditions were manipulated: (a) test length, *J* = 20 or 40, where the 20-item test is within 40 min, and the 40-item test is within 80 min; (b) the number of examinees, *N* = 1, 000 or 2, 000; and (c) the speededness level, low speededness level (LSL) or high speededness level (HSL). The speededness level is controlled by the intensity parameter λ_*j*_. That is, a larger time intensity parameter corresponds to a longer average testing time. Fully crossing the different values of these four factors yielded eight conditions (two test lengths × two sample sizes × two speededness levels).

**True Values and Prior Distributions**

**Figure 1:**
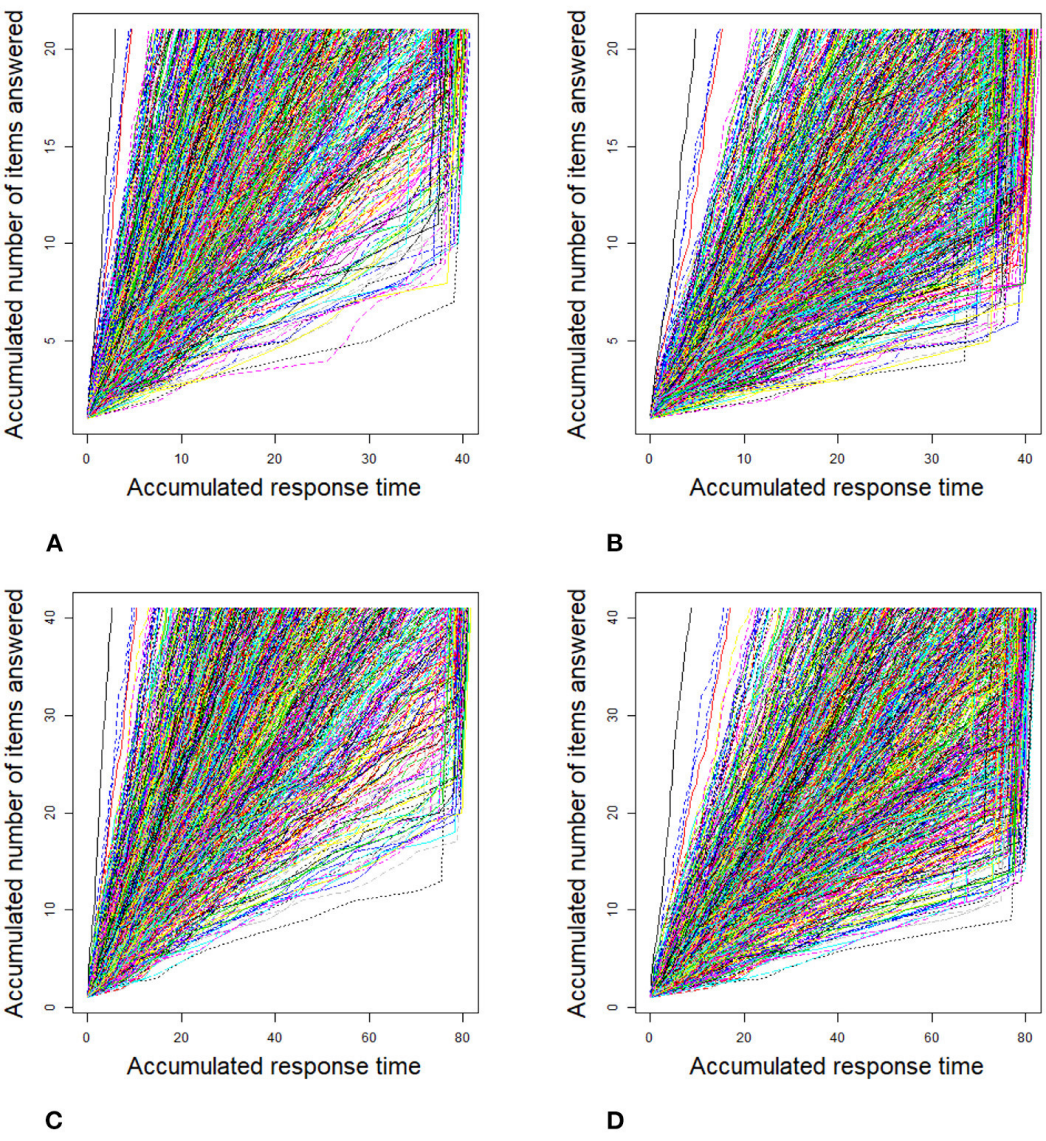
Response time paths for 1,000 examinees at different speededness levels in the simulation study 1. **(A)** items 20 and low speededness, **(B)** items 20 and high speededness, **(C)** items 40 and low speededness, and **(D)** items 40 and high speededness.

**Figure 2:**
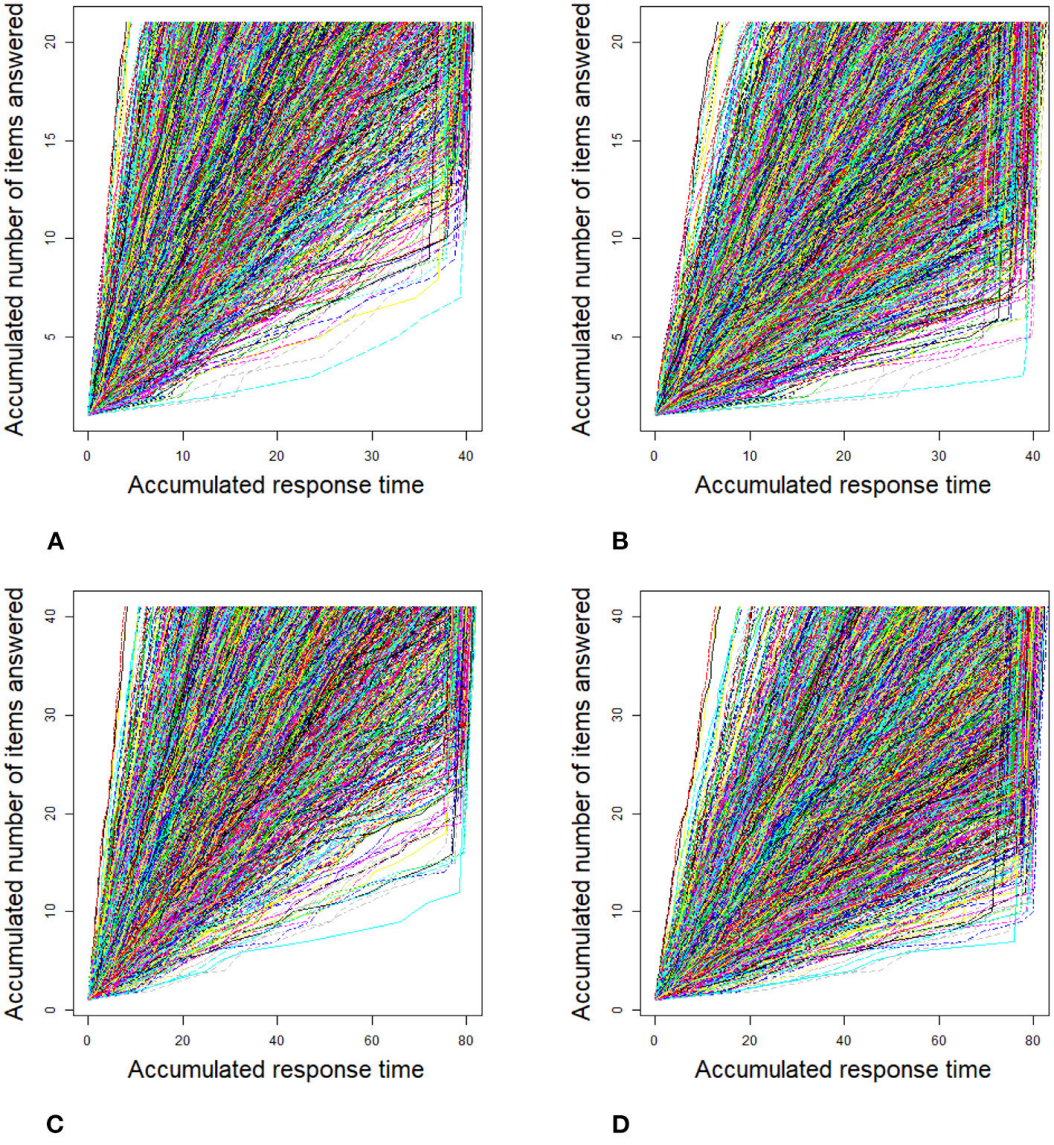
Response time paths for 2,000 examinees at different speededness levels in the simulation study 1. **(A)** items 20 and low speededness, **(B)** items 20 and high speededness, **(C)** items 40 and low speededness, and **(D)** items 40 and high speededness.

**Figure 3:**
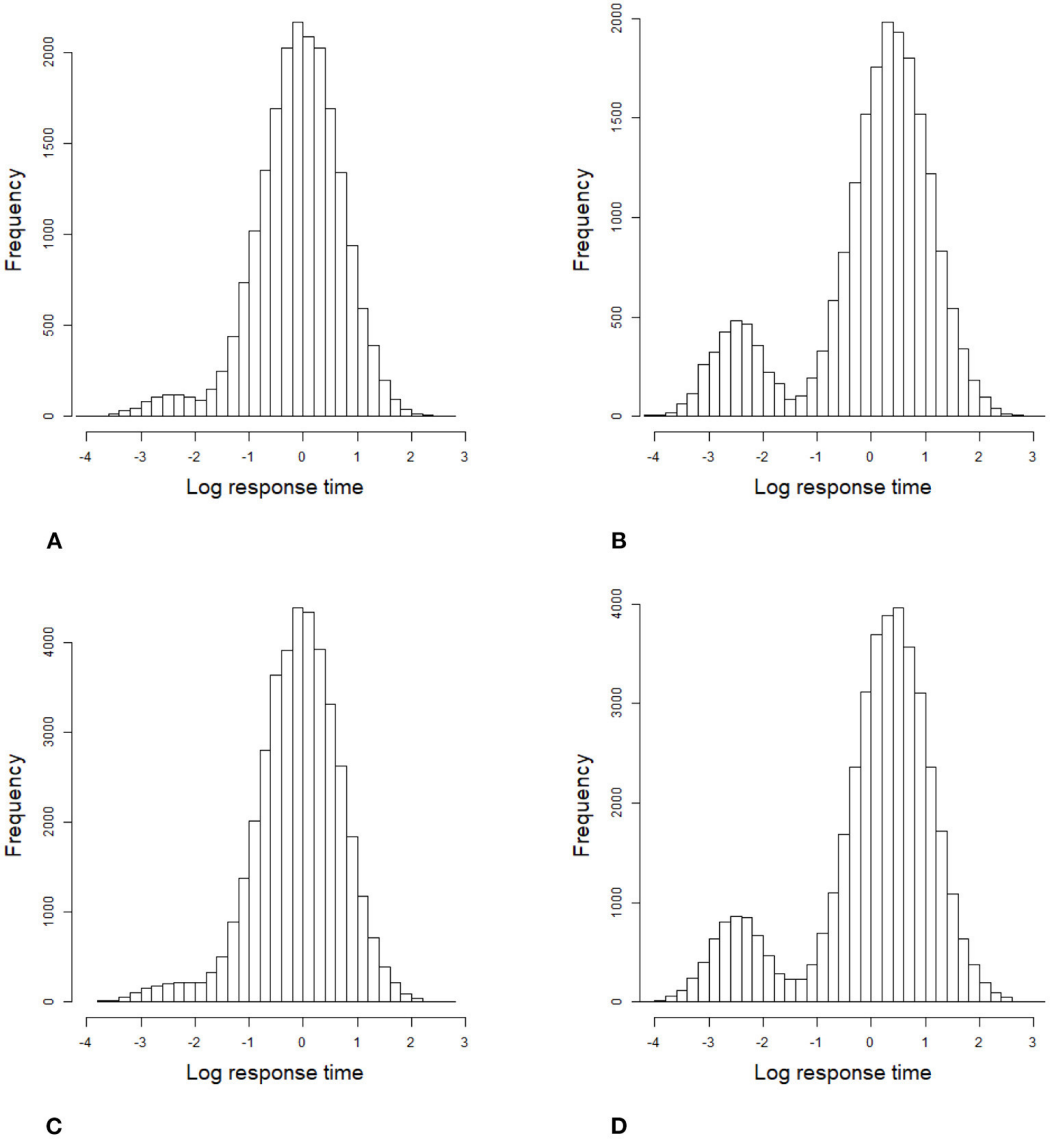
Histogram of 1,000 examinees' response times based on all item–person combinations in the simulation study 1. **(A)** items 20 and low speededness, **(B)** items 20 and high speededness, **(C)** items 40 and low speededness, and **(D)** items 40 and high speededness.

**Figure 4:**
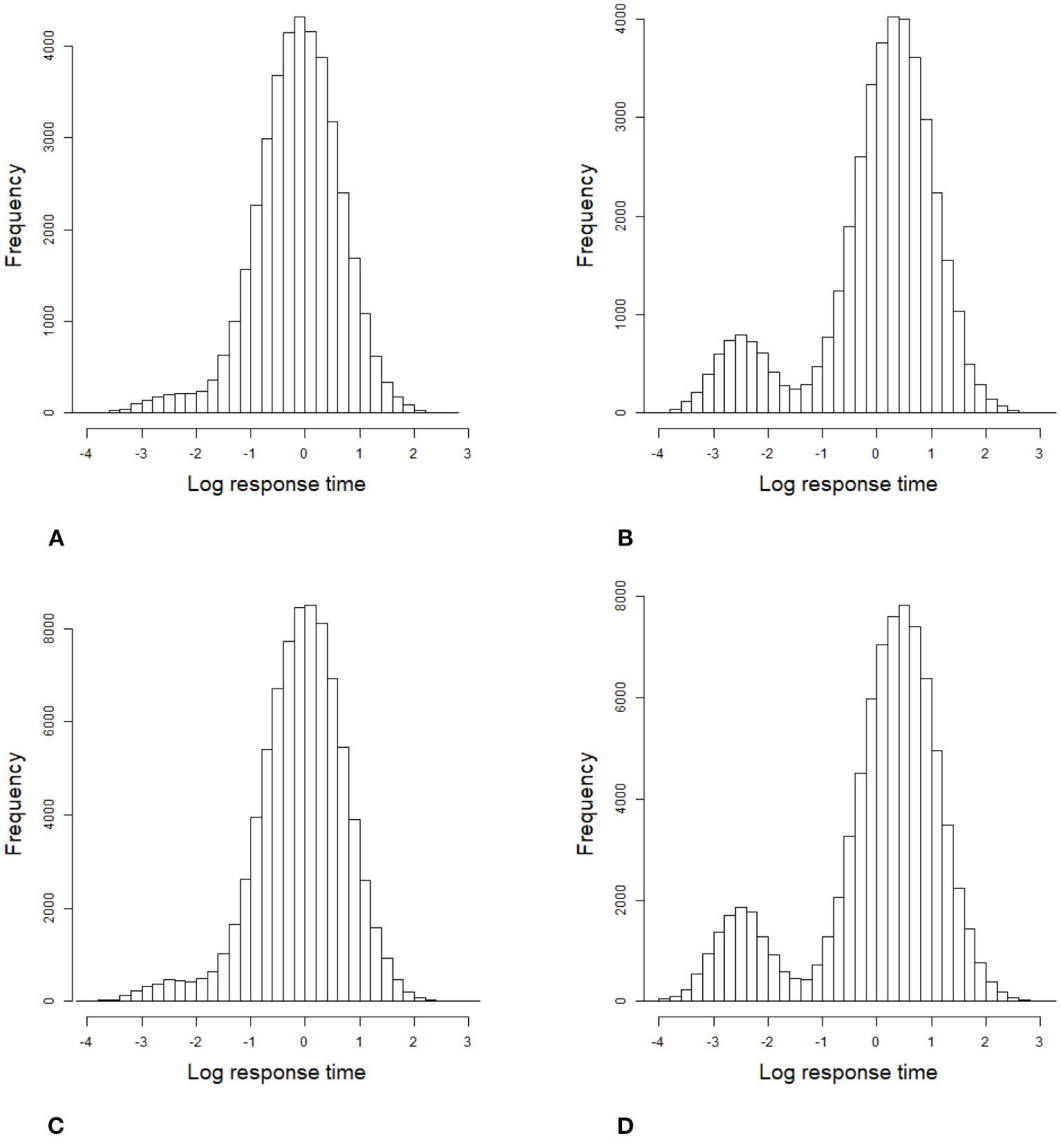
Histogram of 2,000 examinees' response times based on all item–person combinations in the simulation study 1. **(A)** items 20 and low speededness, **(B)** items 20 and high speededness, **(C)** items 40 and low speededness, and **(D)** items 40 and high speededness.

For the 2PL model, true values of item discrimination parameters *a*_*j*_ are generated from a truncated normal distribution, i.e., *a*_*j*_ ~ *N*(0, 1)I(0, +∞), *j* = 1, 2, ..., *J*, where the indicator function I(*A*) takes a value of 1 if *A* is true and 0 if *A* is false. The item difficulty parameters *b*_*j*_ are generated from a standardized normal distribution. For the RT model, the response times of the rapid guessing behavior, *C*_*ij*_, are generated from a log-normal distribution (Wang and Xu, 2015, p. 464), i.e., log*C*_*ij*_ ~ *N*(−2, 0.25). The correct response probability of the rapid guessing behavior, *g*_*j*_, is set to 0.25 for all items (Wang and Xu, 2015). Although the variances of the RT model, $\sigma_{j}^{2},$ can vary across items in the process of model setting and algorithm implementation, for convenience, we assume that the variance of the RT model, $\sigma_{j}^{2}$, is set to 0.5 for all items. We controlled the speededness level by adjusting the time intensity parameter, that is, low speededness distribution λ ~ *U*(−0.25, 0.25) and high speededness distribution λ ~ *U*(0.25, 0.75). The proportion of examinees who could not finish a test within the allocated time is shown in Table 1. The proportion of items that were answered by guessing is also shown in Table 1. For the population distribution of person parameters, the ability and speed parameters (θ, τ)′ were generated from a bivariate normal distribution with mean vector (0, 0)′ and covariance matrix $\left(\begin{array}{cc} 1 & 0.5\\ 0.5 & 0.25 \end{array}\right).$ The responses and response times were generated from the 2PL model and log-normal distribution. The following method was used to generate the guessing behavior indicator η_*ij*_. For all items, examinees could finish a given test within the allotted time having η_*ij*_ = 0, where *j* = 1, ..., *J*. Other η_*ij*_ were generated by the following two steps. Assuming that the generated response time data has no time limit for all items, then we replace *T*_*ij*_ with *C*_*ij*_ from the last item backward until the total response time is less than or equal to the allocated time. Therefore, given the eight simulation conditions, the RT paths for the examinees are shown in Figures 1, 2. Figures 3, 4 show the histograms of response times obtained from all item–person combinations. The non-informative priors and hyperpriors for the parameters were chosen as follows: $p\left(a_{j}\right)\sim N\left(0,10^{5}\right)\text{I}\left(0,+\infty \right),$ $p\left(b_{j}\right)\sim N\left(0,10^{5}\right)$, *p*(*g*_*j*_) ~ *Beta*(5, 17), $p\left(\lambda_{j}\right)\sim N\left(0,10^{5}\right)$, *p*(π_*i*_) ~ *Beta*(1, 5), $p\left(\mu_{c}\right)\sim N\left(-3,10^{5}\right),$ $p\left(\sigma_{c}^{2}\right)\sim$*Inv-Gamma*(0.0001, 0.0001), $p\left(\sigma_{\tau }^{2}\right)\sim$ *Inv-Gamma*(0.0001, 0.0001), and σ_θτ_ $\sim U\left(-\sqrt{\sigma_{\tau }^{2}},\sqrt{\sigma_{\tau }^{2}}\right)$, where $\sigma_{\tau }^{2}=1.$ Fifty replications were considered in each simulation condition.

**Table 1:** The proportions of examinees and items in the simulation study 1.

	**No. of items = 20**	
	**No. of examinees 1,000**	**No. of examinees 2,000**
**Item intensity**	**Proportion of examinees who can**	
	**not finish a 20 item test within 40 min**	
λ ~ *U*(−0.25, 0.25)	14.2%	12.3%
λ ~ *U*(0.25, 0.75)	46.6%	40.5%
**Item intensity**	**Proportion of items that are answered**	
	**by rapid guessing**	
λ ~ *U*(−0.25, 0.25)	3.31%	2.88%
λ ~ *U*(0.25, 0.75)	14.86%	12.59%
	**No. of items = 40**	
	**No. of examinees 1,000**	**No. of examinees 2,000**
**Item intensity**	**Proportion of examinees who can not**	
	**finish a 40 item test within 80 min**	
λ ~ *U*(−0.25, 0.25)	13.4%	15.4%
λ ~ *U*(0.25, 0.75)	44.1%	47.9%
**Item intensity**	**Proportion of items that are**	
	**answered by rapid guessing**	
λ ~ *U*(−0.25, 0.25)	3.05%	3.43%
λ ~ *U*(0.25, 0.75)	13.90%	14.61%

**Convergence diagnostics**

In order to evaluate the convergence of parameter estimates, we only considered convergence in the case of minimum sample sizes with HSLs owing to space limitations. That is, the test length was fixed at 20, and the number of examinees was 1,000. Two methods were used to check the convergence of our algorithm: the “eyeball” method to monitor the convergence by visually inspecting the history plots of the generated sequences; and the Gelman–Rubin method (Gelman and Rubin, 1992; Brooks and Gelman, 1998).

**Figure 5:**
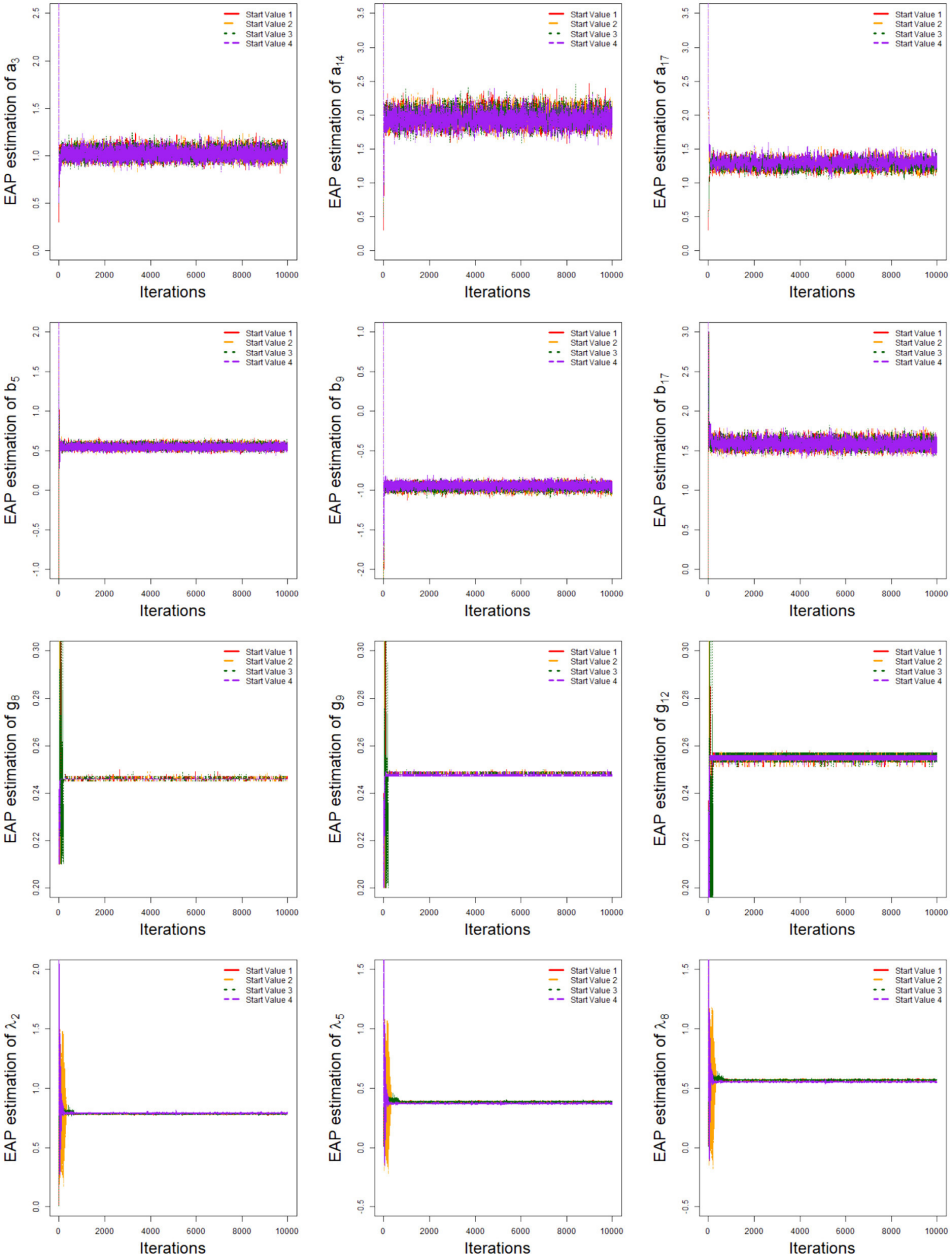
The trace plots of three randomly selected items for the simulation study 1.

**Figure 6:**
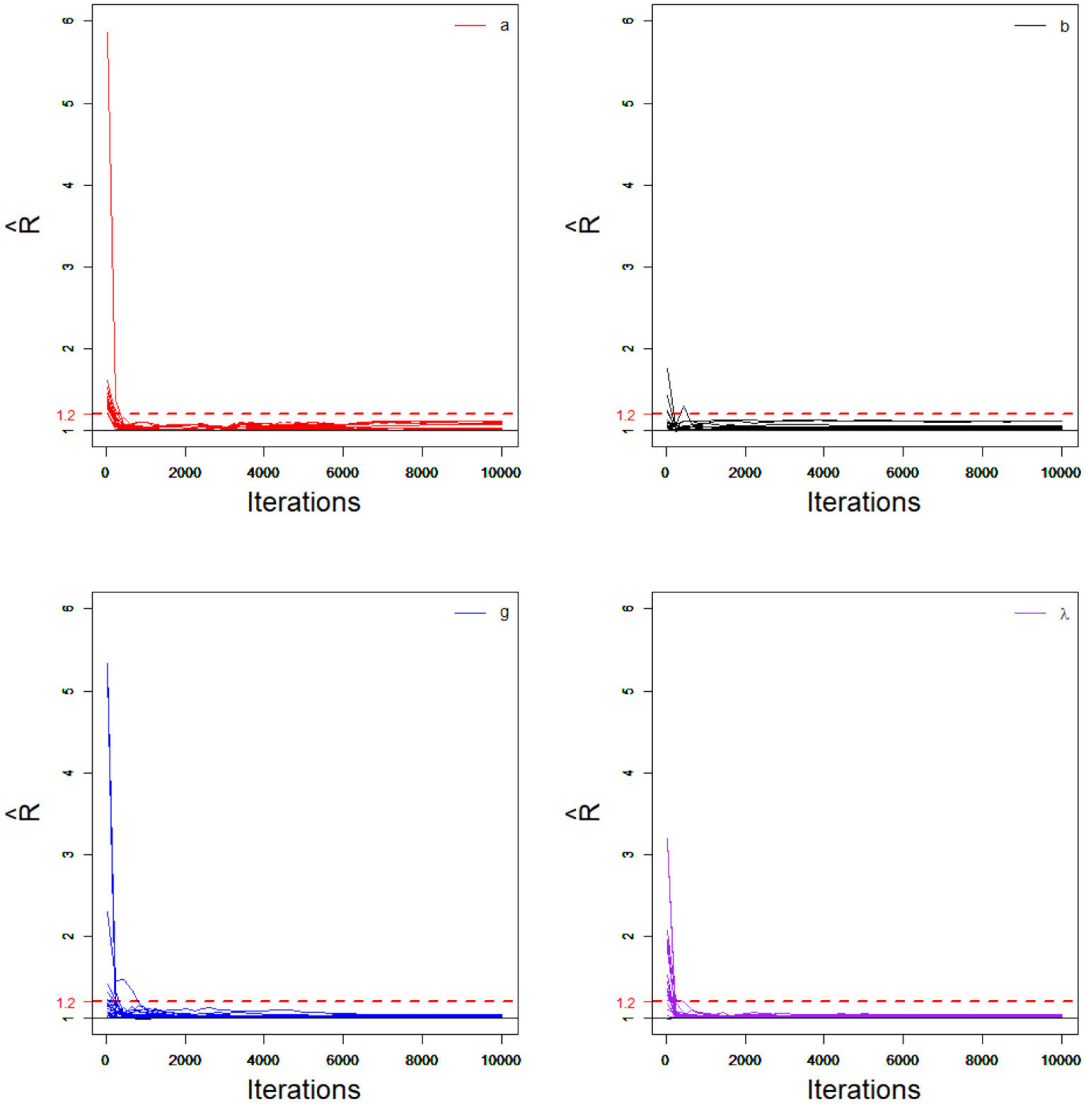
The trace plots of $\hat{R}$ for item parameters in the simulation study 1.

The convergence of the Bayesian algorithm was checked by monitoring the trace plots of the parameters for consecutive sequences of 20,000 iterations. The first 10,000 iterations were set as the burn-in period. As an illustration, four chains started at overdispersed starting values were run for each replication. The trace plots of item parameters randomly selected are shown in Figure 5. In addition, the potential scale reduction factor (PSRF; Brooks and Gelman, 1998) values for all item parameters are shown in Figure 6. We found that the PSRF values for all parameters were less than 1.2, which ensured that all chains converged as expected.

## 5. Results

As shown in Table 2, the bias was between 0.0098 and 0.1411 for the discrimination parameters ***a***, between −0.0335 and 0.0010 for the difficulty parameters ***b***, between −0.0206 and 0.0115 for the rapid guessing parameters ***g***, between −0.0271 and 0.0386 for the time intensity parameters ***λ***, between −0.0105 and 0.0314 for the time discrimination parameters ***σ***^2^, between 0.0196 and 0.0313 for the ability parameters **θ**, between 0.0058 and 0.0377 for the speed parameters **τ**, between −0.0259 and 0.0202 for the μ_*c*_, between −0.0373 and 0.0136 for the $\sigma_{c}^{2}$, between −0.0671 and −0.0102 for the σ_θτ_, and between −0.0201 and 0.0056 for the $\sigma_{\tau }^{2}$. In addition, the MSE was between 0.0125 and 0.0413 for the discrimination parameters ***a***, between 0.0041 and 0.0138 for the difficulty parameters ***b***, between 0.0009 and 0.0026 for the rapid guessing parameters ***g***, between 0.0001 and 0.0017 for the time intensity parameters ***λ***, between 0.0001 and 0.0005 for the time discrimination parameters **σ**^2^, between 0.0873 and 0.1920 for the ability parameters **θ**, between 0.0068 and 0.0693 for the speed parameters **τ**, between 0.0000 and 0.0007 for the μ_*c*_, between 0.0000 and 0.0009 for the $\sigma_{c}^{2}$, between 0.0010 and 0.0045 for the σ_θτ_, and between 0.0002 and 0.0009 for the $\sigma_{\tau }^{2}$. In summary, the Pólya–gamma Gibbs sampling algorithm provides accurate estimates of the parameters for various numbers of examinees and items.

**Table 2:** Evaluating the accuracy of parameters based on mixture model in simulation study 1.

	***N*** = 1, 000, ***J*** = 20		***N*** = 1, 000, ***J*** = 40		***N*** = 2, 000, ***J*** = 20		***N*** = 2, 000, ***J*** = 40	
	**LSL**	**HSL**	**LSL**	**HSL**	**LSL**	**HSL**	**LSL**	**HSL**
**Bias**					
***a***	0.0320	0.0814	0.0098	0.0291	0.1002	0.1411	0.0253	0.0472
***b***	–0.0149	–0.0162	–0.0252	–0.0335	–0.0203	–0.0194	0.0010	–0.0030
***g***	–0.0136	–0.0203	–0.0193	–0.0166	–0.0005	–0.0022	–0.0206	0.0115
***λ***	0.0195	–0.0169	0.0386	0.0160	0.0077	–0.0271	0.0152	–0.0100
**σ** ^2^	–0.0105	0.0058	–0.0062	–0.0041	–0.0092	0.0123	–0.0080	0.0314
**θ**	0.0268	0.0295	0.0210	0.0220	0.0286	0.0313	0.0196	0.0260
**τ**	0.0214	0.0137	0.0377	0.0218	0.0098	0.0058	0.0168	0.0152
μ_*c*_	–0.0259	0.0092	–0.0108	0.0078	–0.0226	0.0069	0.0041	0.0202
$\sigma_{c}^{2}$	–0.0373	0.0132	–0.0371	0.0115	–0.0371	0.0063	–0.0331	0.0136
σ_θτ_	–0.0474	–0.0671	–0.0373	–0.0501	–0.0324	–0.0552	–0.0119	–0.0102
$\sigma_{\tau }^{2}$	–0.0182	–0.0201	–0.0049	–0.0103	–0.0057	–0.0054	0.0037	0.0056
**MSE**					
***a***	0.0252	0.0355	0.0287	0.0375	0.0413	0.0527	0.0125	0.0167
***b***	0.0071	0.0085	0.0105	0.0138	0.0084	0.0108	0.0041	0.0058
***g***	0.0026	0.0018	0.0014	0.0011	0.0013	0.0010	0.0015	0.0009
***λ***	0.0007	0.0011	0.0017	0.0006	0.0001	0.0013	0.0003	0.0015
**σ** ^2^	0.0001	0.0004	0.0001	0.0002	0.0001	0.0005	0.0001	0.0003
**θ**	0.1587	0.1841	0.0943	0.1107	0.1711	0.1920	0.0873	0.1155
**τ**	0.0133	0.0557	0.0080	0.0141	0.0148	0.0693	0.0068	0.0099
μ_*c*_	0.0006	0.0000	0.0001	0.0000	0.0005	0.0000	0.0000	0.0007
$\sigma_{c}^{2}$	0.0003	0.0001	0.0006	0.0001	0.0005	0.0000	0.0009	0.0001
σ_θτ_	0.0022	0.0045	0.0014	0.0025	0.0010	0.0030	0.0015	0.0026
$\sigma_{\tau }^{2}$	0.0003	0.0004	0.0002	0.0005	0.0002	0.0006	0.0005	0.0009

*Note that the Bias and MSE denote the average Bias and MSE for the interested parameters. **a** represents all discrimination parameters, **b** represents all difficulty parameters, **g** represents all rapid guessing parameters, ***λ*** represents all time intensity parameters, **σ**^2^ represents all time discrimination parameters, **θ** represents all ability parameters, and **τ** represents all speed parameters*.

### 5.1. Simulation 2

In this simulation study, we focus on the model fitting data for the mixture model and non-mixture model based on different simulation conditions from the perspective of Bayesian model assessment. Two Bayesian model assessment tools, DIC and LPML, are used to identify the true models.

**Simulation Designs**

For purposes of illustration, the numbers of examinees and items were fixed at 1,000 and 40, respectively. The true value settings for the item parameters in the 2PLIRT model and response time model were the same as in simulation study 1. The first factor is the correlation coefficient. Three correction coefficients ρ_θτ_ were considered in this simulation. That is, (1) ρ_θτ_ = 0.3 (θ and τ have weak correlation; WC); (2) ρ_θτ_ = 0.8 (θ and τ have a strong correlation; SC). Furthermore, the true values of θ and τ can be drawn from a bivariate normal distribution with mean vector **0** and covariance matrix $\left(\begin{array}{cc} 1\; & \rho_{\theta \tau }\\ \rho_{\theta \tau }\; & 1 \end{array}\right)$. The second factor is the speededness level, which was varied by adjusting the time intensity parameter λ: (1) LSL, λ ~ *U*(−0.25, 0.25); (2) HSL, λ ~ *U*(0.25, 0.75). The third factor is the choice of fitting model: (1) mixture model; (2) non-mixture model (hierarchical structure model of van der Linden, 2007). Based on the abovementioned test conditions, the item responses and response time data were respectively generated from the 2PLIRT model and response time model. Therefore, the true models and the fitted models were designed as follows.

- True model, i.e., mixture model with WC (ρ_θτ_ = 0.3)⊕LSL **vs**. fitted model, i.e., mixture model with WC (ρ_θτ_ = 0.3)⊕LSL, and non-mixture model with WC (ρ_θτ_ = 0.3)⊕LSL.
- True model, i.e., mixture model with SC (ρ_θτ_ = 0.8 )⊕LSL **vs**. fitted model, i.e., mixture model with SC (ρ_θτ_ = 0.8)⊕LSL, and non-mixture model with SC (ρ_θτ_ = 0.8)⊕LSL.
- True model, i.e., mixture model with WC (ρ_θτ_ = 0.3)⊕HSL **vs**. fitted model, i.e., mixture model with WC (ρ_θτ_ = 0.3)⊕HSL, and non-mixture model with WC (ρ_θτ_ = 0.3)⊕HSL.
- True model, i.e., mixture model with SC (ρ_θτ_ = 0.8 )⊕HSL **vs**. fitted model, i.e., mixture model with SC (ρ_θτ_ = 0.8)⊕HSL, and non-mixture model with SC (ρ_θτ_ = 0.8)⊕HSL.
- True model, i.e., non-mixture model with WC (ρ_θτ_ = 0.3)⊕LSL **vs**. fitted model, i.e., mixture model with WC (ρ_θτ_ = 0.3)⊕LSL, and non-mixture model with WC (ρ_θτ_ = 0.3)⊕LSL.
- True model, i.e., non-mixture model with SC (ρ_θτ_ = 0.8 )⊕LSL **vs**. fitted model, i.e., mixture model with SC (ρ_θτ_ = 0.8)⊕LSL, and non-mixture model with SC (ρ_θτ_ = 0.8)⊕LSL.
- True model, i.e., non-mixture model with WC (ρ_θτ_ = 0.3)⊕HSL **vs**. fitted model, i.e., mixture model with WC (ρ_θτ_ = 0.3)⊕HSL, and non-mixture model with WC (ρ_θτ_ = 0.3)⊕HSL.
- True model, i.e., non-mixture model with SC (ρ_θτ_ = 0.8 )⊕HSL **vs**. fitted model, i.e., mixture model with SC (ρ_θτ_ = 0.8)⊕HSL, and non-mixture model with SC (ρ_θτ_ = 0.8)⊕HSL.

The priors of parameters were also the same as those used in simulation 1. That is, the non-informative priors were used in this simulation study. To implement the MCMC sampling algorithm, chains of length 10,000 with an initial burn-in period of 20,000 were chosen. There were 50 replications for each simulation condition. The PSRF (Brooks and Gelman, 1998) values for all item and person parameters for each simulation condition were less than 1.2.

**Results**

As shown in Tables 3, 4, regardless of whether the speededness levels were low or high, and whether the correlation coefficients were weak (ρ_θτ_ = 0.3) or strong (ρ_θτ_ = 0.8), both Bayesian model assessment criteria could accurately identify the true models when the data were generated from the mixture models and non-mixture models. More specifically, under the LSL and WC conditions, when the mixture model was the true model, the mixture model fitted the data better, as expected. The median DIC of the mixture model (185007.092) was smaller than that of the non-mixture model (201335.596), and the median LPML of the mixture model (−91302.451) was larger than that of the non-mixture model (−103871.796). Similarly, under the HSL and SC conditions, when the mixture model was the true model, the mixture model also fitted the data best. The differences in the medians of DIC and LPML between the mixture model and non-mixture model were −16705.267 and 6977.566, respectively. In addition, under the LSL and WC conditions, when the non-mixture model was the true model, the non-mixture model fitted the data better. The median DIC of the non-mixture model (188057.725) was smaller than that of the mixture model (192051.824), and the median LPML of the non-mixture model (−93222.498) was larger than that of the mixture model (−95204.235). Similarly, under the HSL and SC conditions, when the non-mixture model was the true model, the mixture model also fitted the data better. The differences in the medians of DIC and LPML between the non-mixture model and mixture model were −4016.200 and 1943.389, respectively. Refer to Tables 3, 4 for more detailed results of the model assessment. In summary, the Bayesian assessment criteria were effective for identifying the true models and could, thus, be used in the subsequent real data study.

**Table 3:** The results of Bayesian model assessment in simulation study 2.

**Low speededness level (LSL)**					
	**Fitted model**			**Mixture model with WC**	**Non-mixture model with WC**
			*Q*_1_	183970.082	200906.367
	Mixture model	DIC	Median	185007.092	201335.596
True	with WC		*Q*_3_	185472.819	201700.856
model	(ρ_θτ_ = 0.3)		*Q*_1_	–91433.366	–103949.160
		LPML	Median	–91302.451	–103871.796
			*Q*_3_	–91095.166	–103782.198
**Low speededness level (LSL)**					
	**Fitted model**			**Mixture model with SC**	**Non-mixture model with SC**
			*Q*_1_	182423.016	200490.494
	Mixture model	DIC	Median	182806.907	200960.661
True	with SC		*Q*_3_	183285.554	201204.742
Model	(ρ_θτ_ = 0.8)		*Q*_1_	–91270.116	–103687.867
		LPML	Median	–91213.797	–103584.228
			*Q*_3_	–91100.563	–103419.208
**High speededness level (HSL)**					
	**Fitted model**			**Mixture model with WC**	**Non-mixture model with WC**
			*Q*_1_	159487.663	175985.981
	Mixture model	DIC	Median	159985.584	176499.862
True	with WC		*Q*_3_	161227.782	176989.732
Model	(ρ_θτ_ = 0.3)		*Q*_1_	–80685.663	–87906.257
		LPML	Median	–80474.893	-87782.508
			*Q*_3_	–80332.172	–87673.533
**High speededness level (HSL)**					
	**Fitted model**			**Mixture model with SC**	**Non-mixture model with SC**
			*Q*_1_	159235.762	175815.800
	Mixture model	DIC	Median	159629.846	176335.113
True	with SC		*Q*_3_	160570.239	176859.457
Model	(ρ_θτ_ = 0.8)		*Q*_1_	–80840.626	–87917.891
		LPML	Median	–80736.678	–87714.244
			*Q*_3_	–80570.342	–87638.130

*Note that the mixture model is the model in Section 2. The non-mixture model is the hierarchical structure model in van der Linden (2007)*.

**Table 4:** The results of Bayesian model assessment in simulation study 2.

**Low speededness level (LSL)**					
	**Fitted**			**Mixture model**	**Non-mixture model**
	**model**			**with WC**	**with WC**
			*Q*_1_	191642.341	187822.030
	Non-mixture model	DIC	Median	192051.824	188057.725
True	with WC		*Q*_3_	192465.323	188289.444
Model	(ρ_θτ_ = 0.3)		*Q*_1_	–95287.618	–93306.447
		LPML	Median	–95204.235	–93222.498
			*Q*_3_	–95146.751	–93168.033
**Low speededness level (LSL)**					
	**Fitted**			**Mixture model**	**Non-mixture model**
	**model**			**with SC**	**with SC**
			*Q*_1_	191663.580	187582.329
	Non-mixture model	DIC	Median	192059.746	187868.073
True	with SC		*Q*_3_	192341.397	187988.874
Model	(ρ_θτ_ = 0.8)		*Q*_1_	–95293.492	–93319.285
		LPML	Median	–95177.928	-93224.461
			*Q*_3_	–95127.793	–93132.479
**High speededness level (HSL)**					
	**Fitted**			**Mixture model**	**Non-mixture model**
	**model**			**with WC**	**with WC**
			*Q*_1_	191880.178	187523.642
	Non-mixture model	DIC	Median	192161.323	187831.945
True	with WC		*Q*_3_	192528.860	188102.832
Model	(ρ_θτ_ = 0.3)		*Q*_1_	–95194.438	–93202.085
		LPML	Median	–95108.402	-93129.144
			*Q*_3_	–94999.978	–93038.260
**High speededness level (HSL)**					
	**Fitted**			**Mixture model**	**Non-mixture model**
	**model**			**with SC**	**with SC**
			*Q*_1_	191396.999	187321.113
	Non-mixture model	DIC	Median	191702.770	187686.570
True	with SC		*Q*_3_	192171.363	187941.382
Model	(ρ_θτ_ = 0.8)		*Q*_1_	–95202.124	–93221.728
		LPML	Median	–95101.015	–93157.626
			*Q*_3_	–95012.373	–93028.099

*Note that the mixture model is the model in Section 2. The non-mixture model is the hierarchical structure model in van der Linden (2007)*.

## 6. Empirical Example

**Figure 7:**
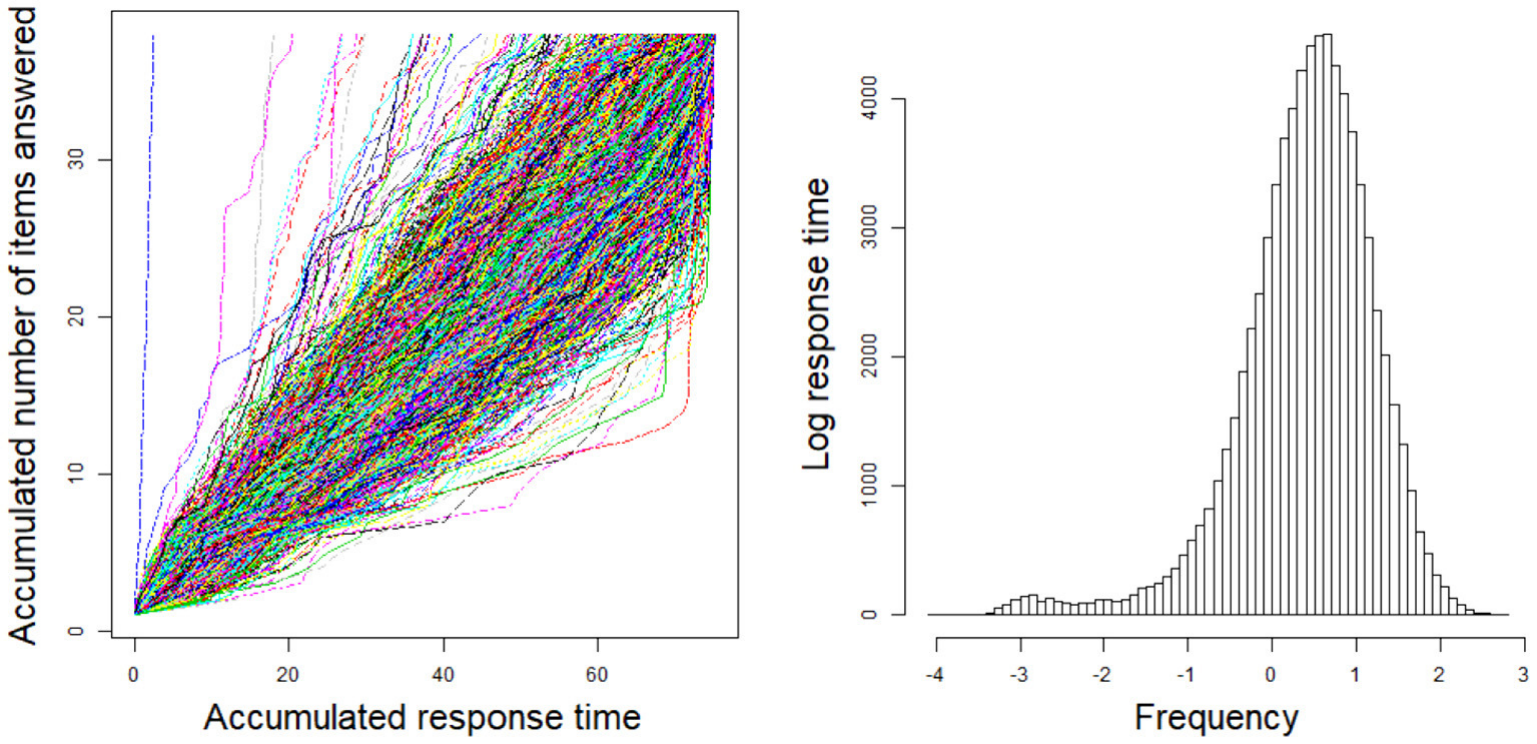
The response time path for the examinees and the histogram of response times obtained from all item-person combinations.

This section presents an application of the mixture model with an empirical example. The data set was from a high-state, large-scale, standardized computerized adaptive test that was previously analyzed by Wang and Xu (2015). The data set included 37 dichotomous items, and the test time was 75 min. The sample size was 2,106. The mixture model and non-mixture model were used to fit the item response and response time data of the 37 dichotomous items. The response time path for the examinees is shown in Figure 7. In addition, Figure 7 shows a histogram of response times obtained from all item–person combinations.

In the Bayesian computation, we used 20,000 MCMC samples after a burn-in of 10,000 iterations to compute all posterior estimates. The convergence of the chains was checked using the PSRF. The PSRF values of all item parameters were less than 1.2. We used the DIC and LPML to fit the mixture model and non-mixture model. The mixture model resulted in a smaller DIC value (350696.11) than the non-mixture model (365690.66), and the LPML of the mixture model (−175027.99) was larger than that of the non-mixture model (−181062.48). This indicates that the mixture model better fitted the data. Based on the results of the model assessment, we used the mixture model to analyze real data in detail.

**Analysis of item parameters**

The estimated results for the discrimination and difficulty parameters are shown in Table 5. As shown in the table, the expected a posteriori (EAP) estimates of the one-item discrimination parameters were greater than 1. This indicated that the items could well distinguish the differences between abilities. The three items with the lowest discrimination were items 4, 34, and 20. The EAP estimates of discrimination parameters for these three items were 0.2000, 0.2024, and 0.2638. In addition, another three items had the lowest EAP estimates of the difficulty parameters, indicating that these items were easier than the other items. These were items 7, 13, and 8. The EAP estimates of *g*_*j*_ had a range of 0.1334 to 0.2945. The EAP estimates of λ_*j*_ had a range of −0.3322 to 0.7634.

**Table 5:** The estimation results of discrimination and difficulty parameter for the real data.

**Para**.		**EAP**		**SD**		**HPDI**	
**a**	**b**	** $\hat{a}$ **	** $\hat{b}$ **	**SD_a_**	**SD_b_**	**HPDI_a_**	**HPDI_b_**
*a*_1_	*b*_1_	0.8182	−0.8649	0.0009	0.0008	[0.7591, 0.8832]	[−0.9234, −0.8073]
*a*_2_	*b*_2_	0.7302	−1.1924	0.0007	0.0015	[0.6809, 0.7862]	[−1.2680, −1.1134]
*a*_3_	*b*_3_	0.4409	−1.2129	0.0003	0.0028	[0.4034, 0.4786]	[−1.3152, −1.1096]
*a*_4_	*b*_4_	0.2000	−1.0279	0.0000	0.0030	[0.1863, 0.2036]	[−1.1353, −0.9183]
*a*_5_	*b*_5_	0.6192	−0.7536	0.0007	0.0010	[0.5652, 0.6715]	[−0.8159, −0.6888]
*a*_6_	*b*_6_	0.5618	−1.0134	0.0005	0.0016	[0.5150, 0.6075]	[−1.0982, −0.9389]
*a*_7_	*b*_7_	0.6946	−1.8531	0.0005	0.0027	[0.6518, 0.7405]	[−1.9656, −1.7591]
*a*_8_	*b*_8_	0.3710	−1.3215	0.0003	0.0042	[0.3350, 0.4046]	[−1.4438, −1.1925]
*a*_9_	*b*_9_	0.5969	−0.6650	0.0008	0.0010	[0.5441, 0.6552]	[−0.7280, −0.6072]
*a*_10_	*b*_10_	0.6228	−0.9849	0.0007	0.0015	[0.5738, 0.6769]	[−1.0609, −0.9129]
*a*_11_	*b*_11_	0.5124	−0.1673	0.0008	0.0004	[0.4601, 0.5719]	[−0.2073, −0.1293]
*a*_12_	*b*_12_	0.7251	−0.8260	0.0008	0.0009	[0.6674, 0.7812]	[−0.8851, −0.7662]
*a*_13_	*b*_13_	0.3342	−1.5034	0.0002	0.0058	[0.3011, 0.3663]	[−1.6613, −1.3594]
*a*_14_	*b*_14_	0.5786	−0.0406	0.0008	0.0003	[0.5179, 0.6319]	[−0.0784, −0.0093]
*a*_15_	*b*_15_	0.3464	−1.2434	0.0003	0.0045	[0.3140, 0.3846]	[−1.3769, −1.1141]
*a*_16_	*b*_16_	1.0816	−0.8625	0.0006	0.0006	[1.0050, 1.1628]	[−0.9109, −0.8092]
*a*_17_	*b*_17_	0.4434	−1.3966	0.0003	0.0035	[0.4070, 0.4823]	[−1.5151, −1.2828]
*a*_18_	*b*_18_	0.6631	−0.2462	0.0010	0.0003	[0.6023, 0.7263]	[−0.2826, −0.2071]
*a*_19_	*b*_19_	0.5072	−0.8406	0.0005	0.0015	[0.4600, 0.5525]	[−0.9186, −0.7620]
*a*_20_	*b*_20_	0.2638	−0.7837	0.0003	0.0042	[0.2251, 0.2972]	[−0.9173, −0.6637]
*a*_21_	*b*_21_	0.5548	−0.7497	0.0006	0.0012	[0.5030, 0.6056]	[−0.8212, −0.6832]
*a*_22_	*b*_22_	0.6791	−0.4723	0.0010	0.0006	[0.6150, 0.7403]	[−0.5235, −0.4273]
*a*_23_	*b*_23_	0.4225	−0.7727	0.0005	0.0019	[0.3803, 0.4670]	[−0.8579, −0.6881]
*a*_24_	*b*_24_	0.7590	−0.5959	0.0011	0.0006	[0.6925, 0.8225]	[−0.6477, −0.5447]
*a*_25_	*b*_25_	0.8798	−0.6894	0.0012	0.0006	[0.8136, 0.9525]	[−0.7414, −0.6393]
*a*_26_	*b*_26_	0.7344	−0.4227	0.0011	0.0005	[0.6680, 0.7990]	[−0.4683, −0.3774]
*a*_27_	*b*_27_	0.5176	−0.6252	0.0007	0.0013	[0.4685, 0.5720]	[−0.6943, −0.5492]
*a*_28_	*b*_28_	0.7185	−0.7225	0.0009	0.0009	[0.6601, 0.7822]	[−0.7846, −0.6619]
*a*_29_	*b*_29_	0.7444	−0.7613	0.0009	0.0009	[0.6797, 0.8024]	[−0.8245, −0.7029]
*a*_30_	*b*_30_	0.5110	−0.4083	0.0007	0.0008	[0.4550, 0.5658]	[−0.4709, −0.3542]
*a*_31_	*b*_31_	0.4307	−0.0292	0.0007	0.0009	[0.3775, 0.4843]	[−0.0911, 0.0303]
*a*_32_	*b*_32_	0.7277	−0.4895	0.0011	0.0008	[0.6624, 0.7954]	[−0.5451, −0.4327]
*a*_33_	*b*_33_	0.5667	0.0485	0.0009	0.0004	[0.5097, 0.6253]	[0.0035, 0.0905]
*a*_34_	*b*_34_	0.2024	−0.7727	0.0000	0.0067	[0.2000, 0.2152]	[−0.9325, −0.6074]
*a*_35_	*b*_35_	0.6925	−0.6144	0.0012	0.0029	[0.6239, 0.7624]	[−0.7182, −0.5086]
*a*_36_	*b*_36_	0.6983	−0.2498	0.0014	0.0064	[0.6228, 0.7744]	[−0.3874, −0.0890]
*a*_37_	*b*_37_	0.4374	0.4227	0.0017	0.0097	[0.3525, 0.5189]	[0.1555, 0.6958]

*Para. denotes the interest parameters. EAP denotes the expected a priori estimation. SD denotes the standard deviation. HPDI denotes the 95% highest posterior density intervals*.

## 7. Conclusion

In this article, we propose a novel and efficient Bayesian algorithm (Pólya–gamma Gibbs sampling algorithm) based on the auxiliary variables for estimating the mixture hierarchical model. The new algorithm avoids the tedious multidimensional integral operation of the MMLE. Within a fully Bayesian framework, the Pólya–gamma Gibbs sampling algorithm not only avoids the heavy reliance of the traditional Metropolis–Hastings algorithm on the tuning parameters of the proposed distributions for different data sets but also overcomes the disadvantage of the Metropolis–Hastings algorithm being sensitive to step size. However, the computational burden of the Pólya–gamma Gibbs sampling algorithm becomes excessive especially when there are a large number of examinees, the items or the abnormal response and response time data are considered, or a large number MCMC sample size is used. Therefore, it would be desirable to develop a stand-alone R package associated with Fortran software for a more extensive large-scale assessment program.

## Data Availability Statement

The original contributions presented in the study are included in the article/Supplementary Materials, further inquiries can be directed to the corresponding authors.

## Author Contributions

ZZ completed the writing of the article. JZ and JL provided original thoughts. ZZ and JL provided key technical support. All authors contributed to the article and approved the submitted version.

## Funding

This work was supported by the National Natural Science Foundation of China (grant no. 12001091), China Postdoctoral Science Foundations (grant nos. 2021M690587 and 2021T140108), the Fundamental Research Funds for the Central Universities of China (grant no. 2412020QD025), Yili Normal University 2021 Annual Research Project (grant no. 2021YSBS012).

## Conflict of Interest

The authors declare that the research was conducted in the absence of any commercial or financial relationships that could be construed as a potential conflict of interest.

## Publisher's Note

All claims expressed in this article are solely those of the authors and do not necessarily represent those of their affiliated organizations, or those of the publisher, the editors and the reviewers. Any product that may be evaluated in this article, or claim that may be made by its manufacturer, is not guaranteed or endorsed by the publisher.
