# Supplementary material for: Bayesian Analysis of Aberrant Response and Response Time Data
Source: Front Psychol. 2022 Apr 25;13:841372. doi: 10.3389/fpsyg.2022.841372 (PMC9083363; doi:10.3389/fpsyg.2022.841372)
Supplement: Supplementary file 1 [file Presentation_1.pdf]

R Codes for Bayesian Parameter Estimation and Model Assessment

```
####################################################
## generate reponse data
####################################################
#library(MASS)
#library(coda)
library(truncnorm)
library(mvtnorm)
library(BayesLogit)
library(MCMCpack)
library(invgamma)

seed<-12
set.seed(seed)
N<-1000
J<-20
text_time<-40
replication<-25

gj <- rep(0.25,J)
mu_p<-c(0,0)
rou<-0.5
sigma_tau<-0.25
sigma_tau0<-sqrt(sigma_tau)
sig_p<-matrix(c(1,1*rou*sigma_tau0,1*rou*sigma_tau0,sigma_tau),2)
sigma_thtau<-sig_p[1,2]
#sig_p<-matrix(c(1,0.25,0.25,0.25),2)
th_tau     <-mvrnorm(N,mu_p,sig_p)
th <-th_tau[ ,1]
tau <-th_tau[ ,2]

a<-rtruncnorm(J, a=1, b=2.5, mean = 0, sd = 1)
b<-rnorm(J,0,1)
Lambda<-runif(J,0,0.5)

A<-outer(th,a)-matrix(rep(a*b,N),N,byrow=TRUE)
p<-exp(A)/(1+exp(A))

log_t<-outer(rep(1,N),Lambda)-outer(tau,rep(1,J))
sig.tj<-rep(0.25,J)
```

```
Tk1 <- matrix(rnorm(N*J, mean = log_t, sd = sqrt(sig.tj)), nrow=N, ncol=J)
Tk0 <- exp(Tk1)

mu_Cij <- -2.5
sigma_Cij <- 0.25
zeta1 <- matrix(rnorm(N*J, mu_Cij, sqrt(sigma_Cij)), ncol=J)
cij <- exp(zeta1)
    tij_cum <- t(apply(Tk0, 1, cumsum))
    tij_cum_0 <- tij_cum[which(apply(Tk0, 1, sum)>text_time),]
    tij_cum_0[tij_cum_0>text_time] <- 0
    tij_cum_0[tij_cum_0 != 0] <- 1
    tij_cum_00 <- matrix(rep(1,N*J),ncol=J)
    tij_cum_00[which(apply(Tk0, 1, sum)>text_time),] <- tij_cum_0
    tij_cum_11 <- 1 - tij_cum_00
Tk <- Tk0*tij_cum_00 + cij*tij_cum_11
T.log<-log(Tk)
eta <- tij_cum_11
eta1 <- tij_cum_11

    p_eta<-matrix(rbinom(N*J, size=1, prob=0.25), ncol=J)
    p_eta<-p_eta*eta

resp<-list()
J_mindiff<-which(b==min(b))
J_maxdiff<-which(b==max(b))
for(k in 1:replication)
{
    R<-matrix(runif(N*J,0,1),N,J)
    resp[[k]]<-ifelse(R<=p,1,0)
    A<-apply(resp[[k]],1,sum)
    is_0<-which(A==0)
    is_1<-which(A==J)
    resp[[k]][is_0,J_mindiff]=1
    resp[[k]][is_1,J_maxdiff]=0
    resp[[k]]<-resp[[k]]*(1-eta) + p_eta
}


####################################################
## DIC LPML
####################################################

log_lik<-function(a,b,th,tau,La,pai,g,eta,mu.c,sig.c,sig.tj,Y,T.log)
{
```

```
        N<-nrow(Y)
        J<-ncol(Y)

    beta.tau <- outer(rep(1,N),La) - outer(tau,rep(1,J))
    paii <- outer(pai,rep(1,J))
    pai.g1 <- outer(pai,g)
    pai.g2 <- outer(pai,(1-g))
    h <- matrix(pnorm(T.log, mu.c, sig.c), ncol=J)
    f <- matrix(pnorm(T.log, beta.tau, sig.tj), ncol=J)
        A<-outer(th,a)-matrix(rep(a*b,N),N,byrow=TRUE)
        p<-1/(1+exp(-A))
        log_0 <- pai.g1*log(h)*eta*Y + pai.g2*log(h)*eta*(1-Y) +
                  (1-paii)*log(p*f)*(1-eta)*Y + (1-paii)*log((1-p)*f)*(1-eta)*(1-Y)
        log_0[is.na(log_0)==1]=0
        return(log_0)
}

DIC<-function(a_k,b_k,th_k,tau_k,lambda_k,pai_k,g_k,eta_k,mu.c_k,sig.c_k,sig.tj_k,Y,T.log)
{
        M<-ncol(a_k)
        H<-rep(NA,M)
        for(m in 1:M)
        {
                Q<-log_lik(a_k[,m],b_k[,m],th_k[,m],tau_k[,m],lambda_k[,m],pai_k[,m],
                           g_k[,m],eta_k[,,m],mu.c_k[m],sig.c_k[m],sig.tj_k[,m],Y,T.log)
                H[m]<-sum(Q, na.rm=TRUE)
        }
        D_hat<- -2*max(H)
        D_bar<- -2*mean(H)
        P_D<-D_bar-D_hat
        DIC<-D_hat+2*P_D
        return(DIC)
}

LPML<-function(a_k,b_k,th_k,tau_k,lambda_k,pai_k,g_k,eta_k,mu.c_k,sig.c_k,sig.tj_k,Y,T.log)
{
        M<-ncol(a_k)
        N<-nrow(Y)
        J<-ncol(Y)
        T_ij<-matrix(NA,M,N*J)
        for(m in 1:M)
        {
                H<-log_lik(a_k[,m],b_k[,m],th_k[,m],tau_k[,m],lambda_k[,m],pai_k[,m],
                           g_k[,m],eta_k[,,m],mu.c_k[m],sig.c_k[m],sig.tj_k[,m],Y,T.log)
```

```
            T_ij[m,]<-as.vector(-H)
        }
        Tij_max<-apply(T_ij,2,max)
        Q<-T_ij-matrix(rep(Tij_max,M),M,byrow=TRUE)
        Q<-exp(Q)
            log_CPO<- -log(colMeans(Q))-Tij_max
        LPML<-sum(log_CPO)
        return(LPML)
}

##################################################
## estimate
##################################################

rPG <- function(a,b,th)
{
        N<-length(th)
        J<-length(a)

        A <- outer(th,a) - outer(rep(1,N),a*b)
        w <- matrix(rpg(N*J, 1, A), nrow=N, ncol=J)

        return(w)
}

rz<-function(a,b,th,w,Y)
{
        N<-length(th)
        J<-length(a)

        A<-outer(th,a) - outer(rep(1,N),a*b)
        x0<-outer(rep(1,N),rep(-10,J))
        b<-outer(rep(1,N),b)
        x2<-outer(rep(1,N),rep(10,J))
        z1<-matrix(rtruncnorm(1, a=x0, b=b, mean = A, sd = sqrt(1/w)),ncol=J)
        z1<-ifelse(Y==0,z1,0)
        z2<-matrix(rtruncnorm(1, a=b, b=x2, mean = A, sd = sqrt(1/w)),ncol=J)
        z2<-ifelse(Y==1,z2,0)
        zb<-z1+z2

        return(zb)
}

ra<-function(b,th,eta,mu.a,sig.a,w,Y)
```

```
{
    J<-ncol(Y)
    N<-nrow(Y)

    th.b <- outer(th,rep(1,J)) - outer(rep(1,N),b)
    eta1 <- 1-eta
    th.b <- ifelse(eta1!=0,th.b,NA)
    K <- (Y-1/2)*th.b
    sig <- 1/(1/sig.a + apply((th.b^2)*w,2,sum,na.rm=TRUE))
    mu    <- sig*(mu.a/sig.a + apply(K,2,sum,na.rm=TRUE))
    a      <- rtruncnorm(1, a=0.5, b=3, mean = mu, sd = sig)

  return(a)
}

rb<-function(a,th,eta,mu.b,sig.b,w,Y)
{
    J<-ncol(Y)
    N<-nrow(Y)

    A <- outer(rep(1,N),a)
    eta1 <- 1-eta
    A <- ifelse(eta1!=0,A,NA)
    K <- 2*outer(th,a^2)*w - 2*Y*A + A
    sig <- 1/(1/sig.b + apply((A^2)*w,2,sum,na.rm=TRUE))
    mu    <- sig*(mu.b/sig.b + apply(K,2,sum,na.rm=TRUE)/2)
    b      <- rtruncnorm(1, a=-3, b=3, mean = mu, sd = sig)

    return(b)
}

rth<-function(a,b,eta,mu_th,sig_th,z,w,Y)
{
    J<-ncol(Y)
    N<-nrow(Y)

    A <- outer(rep(1,N),a)
    eta1 <- 1-eta
    A <- ifelse(eta1!=0,A,NA)
    z_b <- z + outer(rep(1,N),a*b)
    z_b <- ifelse(eta1!=0,z_b,NA)
    sig <- 1/(1/sig_th + apply((A^2)*w,1,sum,na.rm=TRUE))
    mu    <- sig*(mu_th/sig_th^2 + apply(A*z_b*w,1,sum,na.rm=TRUE))
    th    <- rnorm(N, mean = mu, sd = sig)
```

```
  return(th)
}

reta<-function(a,b,th,tau,La,pai,g,mu.c,sig.c,sig.tj,Y,T.log)
{
    J<-ncol(Y)
    N<-nrow(Y)

    A<-outer(th,a) - outer(rep(1,N),a*b)
    p<-exp(A)/(1+exp(A))
    h<-matrix(dnorm(T.log, mean = mu.c, sd = sig.c), ncol=J)
    mu.tj<-outer(rep(1,N),La)-outer(tau,rep(1,J))
    sig.tj0<-outer(rep(1,N),sig.tj)
    f<-matrix(dnorm(T.log, mean = mu.tj, sd = sig.tj0), ncol=J)
    g0<-outer(rep(1,N),g)
    pai0<-outer(pai,rep(1,J))

    p1 <- (pai0*g0*h)/(pai0*g0*h + (1-pai0)*p*f)
    p1 <- ifelse(is.na(p1)==1,0.5,p1)
    p0 <- (pai0*(1-g0)*h)/(pai0*(1-g0)*h + (1-pai0)*(1-p)*f)
    p0 <- ifelse(is.na(p0)==1,0.5,p0)

    eta1 <- ifelse(p1>runif(1),1,0)
    eta1 <- ifelse(Y==1,eta1,30)
    eta0 <- ifelse(p0>runif(1),1,0)
    eta0 <- ifelse(Y==0,eta0,30)
    eta <- eta1 + eta0
    eta <- ifelse(eta==30,0,eta)
    eta <- ifelse(eta>30,1,eta)

    eta[which(rowSums(exp(T.log))<5*text_time/6),]<-0

  return(eta)
}

rpai<-function(eta,Y)
{
    J<-ncol(Y)
    N<-nrow(Y)

        iota3<-1 + rowSums(eta)
        iota4<-5 + J - rowSums(eta)
```

```
        pai<-rbeta(N,iota3,iota4)
        return(pai)
}

rgj<-function(eta,Y)
{
    J<-ncol(Y)
    N<-nrow(Y)

        gj <- rbeta(J, 5+colSums(Y*eta), 17+colSums(eta)-colSums(Y*eta))
        return(gj)
}

rtau<-function(th,eta,La,sig.tau,sig.th.tau,sig.tj,T.log)
{
    J<-ncol(T.log)
    N<-nrow(T.log)

    sig.tj<-outer(rep(1,N),sig.tj)
    La<-outer(rep(1,N),La)
    th<-outer(th,rep(1,J))
    sig <- abs(1/(1/(sig.tau-sig.th.tau^2)+rowSums((1-eta)/sig.tj)))
    mu <- sig*(sig.th.tau*th/(sig.tau-sig.th.tau^2) + rowSums((1-eta)*(La-T.log)/sig.tj))

    tau <- rnorm(N, mean = mu, sd = sig)

  return(tau)

}

rlambda<-function(tau,eta,sig.tj,T.log)
{
    J<-ncol(T.log)
    N<-nrow(T.log)

    sig.tj<-outer(rep(1,N),sig.tj)
    tau<-outer(tau,rep(1,J))
    sig <- 1/(1 + colSums(1-eta))
    mu <- sig*(1 + colSums((1-eta)*(T.log+tau)))

    lambda <- rnorm(J, mean = mu, sd = sig)
  return(lambda)

}
```

```
rsig.tj<-function(eta,La,tau,T.log)
{
    J<-ncol(T.log)
    N<-nrow(T.log)

    La<-outer(rep(1,N),La)
    tau<-outer(tau,rep(1,J))
    v1<-colSums(1-eta)
    w1<-colSums((1-eta)*(T.log-La+tau)^2)
    sig.tj<-rinvgamma(J, 0.0001+v1/2, 0.0001+w1/2)
    sig.tj<-ifelse((1/sig.tj)==0,0.1,sig.tj)

    return(sig.tj)
}

rmu.c<-function(eta,sig.c,T.log)
{
    mu.prior<--2
    sig.prior<-0.25
    sig <- sqrt(sig.c*sig.prior)/sqrt(sig.prior*sum(eta)+sig.c)
    mu <- (mu.prior*sig.c + sig.prior*sum(eta*T.log))/(sig.c + sig.prior*sum(eta))
    mu.c <- rtruncnorm(1, a=-Inf, b=0, mean = mu, sd = sig)
    mu.c <- ifelse(is.na(mu.c)==1,mu.c0,mu.c)

  return(mu.c)
}

rsig.c<-function(eta,sig.c.old,T.log)
{
        sig.c0<-sig.c.old
        sig.cc <- var(T.log[which(eta*T.log!=0)])/2
        sig.ct <- var(as.vector(T.log))/3
        sig.c <- rtruncnorm(1, a=sig.cc, b=0.3, mean=sig.ct, sd=0.1)
        sig.c <- ifelse(is.na(sig.c)==1,sig.c0,sig.c)
        return(sig.c)
}

rsig.th.tau<-function(sig.tt.old,sig.tau,th,tau)
{
    sig.tt0<-sig.tt.old
    s01=0.1
    p01<-2*sig.tau
    sig.tt1<-rtruncnorm(1,a=-p01,b=p01,mean=sig.tt0,sd=s01)
```

```
    q10<-(p01-sig.tt0)/s01
    q20<-(-p01-sig.tt0)/s01
    q11<-(p01-sig.tt1)/s01
    q21<-(-p01-sig.tt1)/s01

    k10<-pnorm(q=q10, mean = 0, sd = 1)
    k20<-pnorm(q=q20, mean = 0, sd = 1)
    k11<-pnorm(q=q11, mean = 0, sd = 1)
    k21<-pnorm(q=q21, mean = 0, sd = 1)

    mu0<-(sig.tt0)*th
    mu1<-(sig.tt1)*th
    sd0<-abs(sig.tau-(sig.tt0)^2)
    sd1<-abs(sig.tau-(sig.tt1)^2)

    p0<-dnorm(tau, mean = mu0, sd = sqrt(sd0))
    p1<-dnorm(tau, mean = mu1, sd = sqrt(sd1))

    ru0<-dunif(sig.tt0, min = 0, max = 1)
    ru1<-dunif(sig.tt1, min = 0, max = 1)

    pro<-sum(log(p1))+log(ru1)+log(k10-k20)-sum(log(p0))-log(ru0)-log(k11-k21)
    choose<-pmin(1,exp(pro))
    sig.th.tau<-ifelse(choose>runif(1),sig.tt1,sig.tt0)

    return(sig.th.tau)
}

rsig.tau<-function(sig.tau.old,sig.th.tau,th,tau)
{
    sig.tau0<-sig.tau.old
    s02=0.1
    p0<-sig.th.tau
    sig.tau1<-rtruncnorm(1,a=p0,b=Inf,mean=sig.tau0,sd=sqrt(s02))

    q10<-(p0-sig.tau0)/s02
    q11<-(p0-sig.tau1)/s02

    k10<-pnorm(q=q10, mean = 0, sd = 1)
    k11<-pnorm(q=q11, mean = 0, sd = 1)

    mu0<-(sig.th.tau)*th
    mu1<-(sig.th.tau)*th
```

```
    sd0<-abs(sig.tau0-(sig.th.tau)^2)
    sd1<-abs(sig.tau1-(sig.th.tau)^2)

    p0<-dnorm(tau, mean = mu0, sd = sqrt(sd0))
    p1<-dnorm(tau, mean = mu1, sd = sqrt(sd1))

    v1<-0.002
    w1<-0.002
    ch0<-dgamma(1/sig.tau0, shape=v1, rate = w1)
    ch1<-dgamma(1/sig.tau1, shape=v1, rate = w1)

    pro<-sum(log(p1))+log(ch1)+log(1-k10)-sum(log(p0))-log(ch0)-log(1-k11)
    choose<-pmin(1,exp(pro))
    sig.tau<-ifelse(choose>runif(1),sig.tau1,sig.tau0)

    return(sig.tau)
}

pg_sample<-function(data,init,n_length,hypara=hypara)
{
        mu.a <- hypara$mu.a
        sig.a<- hypara$sig.a
        mu.b <- hypara$mu.b
        sig.b<- hypara$sig.b
        mu.th <- hypara$mu.th
        sig.th<- hypara$sig.th
        mu.La <- hypara$mu.La
        sig.La<- hypara$sig.La
        Y<-data$Y
        T.log<-data$T.log

        n_student<-nrow(Y)
        n_item<-ncol(Y)

        a_k<-matrix(,n_item,n_length)
        b_k<-matrix(,n_item,n_length)
        th_k<-matrix(,n_student,n_length)
        eta_k<-array(NA,dim=c(n_student,n_item,n_length))
        pai_k<-matrix(,n_student,n_length)
        gj_k<-matrix(,n_item,n_length)
        tau_k<-matrix(,n_student,n_length)
        lambda_k<-matrix(,n_item,n_length)
        sig.tj_k<-matrix(,n_item,n_length)
        mu.c_k<-vector(length=n_length)
```

```
sig.c_k<-vector(length=n_length)
sig.th.tau_k<-vector(length=n_length)
sig.tau_k<-vector(length=n_length)

rownames(a_k)<-paste('a',1:n_item)
colnames(a_k)<-paste('iter',1:n_length)
rownames(b_k)<-paste('b',1:n_item)
colnames(b_k)<-paste('iter',1:n_length)
rownames(th_k)<-paste('th',1:n_student)
colnames(th_k)<-paste('iter',1:n_length)
rownames(pai_k)<-paste('pai',1:n_student)
colnames(pai_k)<-paste('iter',1:n_length)
rownames(gj_k)<-paste('gj',1:n_item)
colnames(gj_k)<-paste('iter',1:n_length)
rownames(tau_k)<-paste('tau',1:n_student)
colnames(tau_k)<-paste('iter',1:n_length)
rownames(lambda_k)<-paste('lambda',1:n_item)
colnames(lambda_k)<-paste('iter',1:n_length)
rownames(sig.tj_k)<-paste('sig.tj',1:n_item)
colnames(sig.tj_k)<-paste('iter',1:n_length)
names(mu.c_k)<-paste('iter',1:n_length)
names(sig.c_k)<-paste('iter',1:n_length)
names(sig.th.tau_k)<-paste('iter',1:n_length)
names(sig.tau_k)<-paste('iter',1:n_length)

a_k[,1]<-init$a0
b_k[,1]<-init$b0
th_k[,1]<-init$th0
eta_k[,,1]<-init$eta0
pai_k[,1]<-init$pai0
gj_k[,1]<-init$gj0
tau_k[,1]<-init$tau0
lambda_k[,1]<-init$lambda0
sig.tj_k[,1]<-init$sig.tj0
mu.c_k[1]<-init$mu.c0
sig.c_k[1]<-init$sig.c0
sig.th.tau_k[1]<-init$sig.th.tau0
sig.tau_k[1]<-init$sig.tau0

for(k in 1:(n_length-1))
{
	if(k%%100==0) print(k)

	w_k <- rPG(a=a_k[,k],b=b_k[,k],th=th_k[,k])
```

```
        z <- rz(a=a_k[,k],b=b_k[,k],th=th_k[,k],w=w_k,Y)

        a_k[,k+1]<-ra(b=b_k[,k],th=th_k[,k],eta=eta_k[,,k],mu.a,sig.a,w=w_k,Y)
        b_k[,k+1]<-rb(a=a_k[,k],th=th_k[,k],eta=eta_k[,,k],mu.b,sig.b,w=w_k,Y)
        th_k[,k+1]<-rth(a=a_k[,k],b=b_k[,k],eta=eta_k[,,k],mu.th,sig.th,z=z,w=w_k,Y)
        th_k[,k+1]<-(th_k[,k+1]-mean(th_k[,k+1]))/sd(th_k[,k+1])

    eta_k[,,k+1]<-reta(a=a_k[,k],b=b_k[,k],th=th_k[,k],tau=tau_k[,k],La=lambda_k[,k],pai=pai_k[,
k],g=gj_k[,k],mu.c=mu.c_k[k],sig.c=sig.c_k[k],sig.tj=sig.tj_k[,k],Y,T.log)
        pai_k[,k+1]<-rpai(eta=eta_k[,,k],Y)
        gj_k[,k+1]<-rgj(eta=eta_k[,,k],Y)
        tau_k[,k+1]<-rtau(th=th_k[,k],eta=eta_k[,,k],La=lambda_k[,k],sig.tau=sig.tau_k[k],
                        sig.th.tau=sig.th.tau_k[k],sig.tj=sig.tj_k[,k],T.log)
        lambda_k[,k+1]<-rlambda(tau=tau_k[,k],eta=eta_k[,,k],sig.tj=sig.tj_k[,k],T.log)
        sig.tj_k[,k+1]<-rsig.tj(eta=eta_k[,,k],La=lambda_k[,k],tau=tau_k[,k],T.log)
        mu.c_k[k+1]<-rmu.c(eta=eta_k[,,k],sig.c=sig.c_k[k],T.log)
        sig.c_k[k+1]<-rsig.c(eta=eta_k[,,k],sig.c.old=sig.c_k[k],T.log)
    sig.th.tau_k[k+1]<-rsig.th.tau(sig.tt.old=sig.th.tau_k[k],sig.tau=sig.tau_k[k],th=th_k[,k],tau=ta
u_k[,k])
    sig.tau_k[k+1]<-rsig.tau(sig.tau.old=sig.tau_k[k],sig.th.tau=sig.th.tau_k[k],th=th_k[,k],tau=tau
_k[,k])
    }

    return(list(a=a_k,b=b_k,th=th_k,eta=eta_k,pai=pai_k,gj=gj_k,tau=tau_k,lambda=lambda_k,
            sig.tj=sig.tj_k,mu.c=mu.c_k,sig.c=sig.c_k,sig.th.tau=sig.th.tau_k,sig.tau=sig.tau_k))

}


##################################################
## main
##################################################

timestart<-Sys.time()
timestart
library(snow)

n_cores <- 25
replay<-25
cl <- makeCluster(n_cores, type="SOCK")

n_length<-10000
n_burn=5000
n_sample=2500
```

```
step=(n_length-n_burn)/n_sample
d<-length(n_burn+step*(1:n_sample))

source('00useful_before_data.R')
source('00data.R')
N
J

func <- function(L)
{
source('00useful_before_data.R')
source('00data.R')
options(digits=4)

n_length=10000
n_burn=5000
n_sample=2500
step=(n_length-n_burn)/n_sample

Y<-resp[[L]]
T.log=T.log

        thh <- rnorm(N,0,1)
        A<-outer(thh,a)
        ww <- matrix(rpg(N*J, 1, A), nrow=N, ncol=J)
        xx0<-outer(rep(1,N),rep(-3,J))
        bb<-outer(rep(1,N),b)
        xx2<-outer(rep(1,N),rep(3,J))
        zz1<-matrix(rtruncnorm(1, a=xx0, b=bb, mean = A, sd = sqrt(1/ww)),ncol=J)
        zz1<-ifelse(Y==0,zz1,0)
        zz2<-matrix(rtruncnorm(1, a=bb, b=xx2, mean = A, sd = sqrt(1/ww)),ncol=J)
        zz2<-ifelse(Y==1,zz2,0)
        z0<-zz1+zz2
        A0<-outer(thh,a) - outer(rep(1,N),a*b)
        w0 <- matrix(rpg(N*J, 1, A0), nrow=N, ncol=J)

eta00<-matrix(rbinom(N*J, size=1, prob=c(rep(0.001,J-5),rep(0.2,5))), ncol=J, byrow=TRUE)

data<-list(Y=Y,T.log=T.log)
init<-list(a0=runif(J,1,2.5),b0=rnorm(J,0,1),th0=rnorm(N,0,1),w0=w0,eta0=eta00,pai0=rep(0.1,N),
gj0=rep(0.01,J),
                tau0=rnorm(N,0,0.2),lambda0=rep(0.1,J),sig.tj0=rep(0.5,J),mu.c0=-3,sig.c0=0.2,
                sig.th.tau0=0.25,sig.tau0=0.5)
hypara=list(mu.a=0,sig.a=10^5,mu.b=0,sig.b=10^5,mu.th=0,sig.th=10^5,mu.La=0,sig.La=1)
```

```
fit<-pg_sample(data,init,n_length,hypara=hypara)

a_k<-fit$a
b_k<-fit$b
th_k<-fit$th
eta_k<-fit$eta
pai_k<-fit$pai
gj_k<-fit$gj
tau_k<-fit$tau
lambda_k<-fit$lambda
sig.tj_k<-fit$sig.tj
mu.c_k<-fit$mu.c
sig.c_k<-fit$sig.c
sig.th.tau_k<-fit$sig.th.tau
sig.tau_k<-fit$sig.tau

a_sample=a_k[,n_burn+step*(1:n_sample)]
b_sample=b_k[,n_burn+step*(1:n_sample)]
th_sample=th_k[,n_burn+step*(1:n_sample)]
eta_sample=eta_k[,,n_burn+step*(1:n_sample)]
pai_sample=pai_k[,n_burn+step*(1:n_sample)]
gj_sample=gj_k[,n_burn+step*(1:n_sample)]
tau_sample=tau_k[,n_burn+step*(1:n_sample)]
lambda_sample=lambda_k[,n_burn+step*(1:n_sample)]
sig.tj_sample=sig.tj_k[,n_burn+step*(1:n_sample)]
mu.c_sample=mu.c_k[n_burn+step*(1:n_sample)]
sig.c_sample=sig.c_k[n_burn+step*(1:n_sample)]
sig.th.tau_sample=sig.th.tau_k[n_burn+step*(1:n_sample)]
sig.tau_sample=sig.tau_k[n_burn+step*(1:n_sample)]

DIC_est<-DIC(a_k=a_sample,b_k=b_sample,th_k=th_sample,tau_k=tau_sample,lambda_k=lambd
a_sample,
             pai_k=pai_sample,g_k=gj_sample,eta_k=eta_sample,mu.c_k=mu.c_sample,
             sig.c_k=sig.c_sample,sig.tj_k=sig.tj_sample,Y,T.log)
LPML_est<-LPML(a_k=a_sample,b_k=b_sample,th_k=th_sample,tau_k=tau_sample,lambda_k=la
mbda_sample,
             pai_k=pai_sample,g_k=gj_sample,eta_k=eta_sample,mu.c_k=mu.c_sample,
             sig.c_k=sig.c_sample,sig.tj_k=sig.tj_sample,Y,T.log)
model_select<-rbind(DIC_est,LPML_est)

eap_eta<-eta_sample[,,n_sample]
eap_eta04<-apply(eta_sample, c(1,2), sum)>=n_sample*0.4
eap_eta05<-apply(eta_sample, c(1,2), sum)>=n_sample*0.5
```

```
eap_eta06<-apply(eta_sample, c(1,2), sum)>=n_sample*0.6

n1<-N*J-sum(eap_eta==eta)
ThresholdLastEta<-n1/(N*J)
n2<-N*J-sum(eap_eta04==eta)
Threshold04<-n2/(N*J)
n3<-N*J-sum(eap_eta05==eta)
Threshold05<-n3/(N*J)
n4<-N*J-sum(eap_eta06==eta)
Threshold06<-n4/(N*J)
Misclassification_rate<-rbind(Threshold04,Threshold05,Threshold06,ThresholdLastEta)

write.csv(model_select,paste0('model_select_dataset_',L,'.csv'))
write.csv(Misclassification_rate,paste0('Misclassification_rate_',L,'.csv'))
write.csv(a_k,paste0('a_k_dataset_',L,'.csv'))
write.csv(a_sample,paste0('a_sample_dataset_',L,'.csv'))
write.csv(b_k,paste0('b_k_dataset_',L,'.csv'))
write.csv(b_sample,paste0('b_sample_dataset_',L,'.csv'))
write.csv(th_k,paste0('th_k_dataset_',L,'.csv'))
write.csv(th_sample,paste0('th_sample_dataset_',L,'.csv'))
write.csv(pai_k,paste0('pai_k_dataset_',L,'.csv'))
write.csv(pai_sample,paste0('pai_sample_dataset_',L,'.csv'))
write.csv(gj_k,paste0('gj_k_dataset_',L,'.csv'))
write.csv(gj_sample,paste0('gj_sample_dataset_',L,'.csv'))
write.csv(tau_k,paste0('tau_k_dataset_',L,'.csv'))
write.csv(tau_sample,paste0('tau_sample_dataset_',L,'.csv'))
write.csv(lambda_k,paste0('lambda_k_dataset_',L,'.csv'))
write.csv(lambda_sample,paste0('lambda_sample_dataset_',L,'.csv'))
write.csv(mu.c_k,paste0('mu.c_k_dataset_',L,'.csv'))
write.csv(mu.c_sample,paste0('mu.c_sample_dataset_',L,'.csv'))
write.csv(sig.c_k,paste0('sig.c_k_dataset_',L,'.csv'))
write.csv(sig.c_sample,paste0('sig.c_sample_dataset_',L,'.csv'))
write.csv(sig.tj_k,paste0('sig.tj_k_dataset_',L,'.csv'))
write.csv(sig.tj_sample,paste0('sig.tj_sample_dataset_',L,'.csv'))
write.csv(sig.th.tau_k,paste0('sig.th.tau_k_dataset_',L,'.csv'))
write.csv(sig.th.tau_sample,paste0('sig.th.tau_sample_dataset_',L,'.csv'))
write.csv(sig.tau_k,paste0('sig.tau_k_dataset_',L,'.csv'))
write.csv(sig.tau_sample,paste0('sig.tau_sample_dataset_',L,'.csv'))

}
timestart
invisible(parLapply(cl, 1:replay, func))
stopCluster(cl)
################ end ##############
```
